# High cell density cultivation by anaerobic respiration

**DOI:** 10.1186/s12934-024-02595-8

**Published:** 2024-11-25

**Authors:** Marte Mølsæter Maråk, Ricarda Kellermann, Linda Liberg Bergaust, Lars Reier Bakken

**Affiliations:** Faculty of Biotechnology, Chemistry and Food Science, Norwegian University for Life Sciences, Ås, Norway

**Keywords:** High cell density cultivation, Denitrification, pH–stat

## Abstract

**Background:**

Oxygen provision is a bottleneck in conventional aerobic high cell density culturing (HCDC) of bacteria due to the low O_2_ solubility in water. An alternative could be denitrification: anaerobic respiration using nitrogen oxides as terminal electron acceptors. Denitrification is attractive because NO_3_^−^ is soluble in water, the end-product (N_2_) is harmless, and denitrification is widespread among bacteria, hence suitable organisms for most purposes can be found. The pH must be controlled by injection of an inorganic acid to compensate for the pH increase by NO_3_^−^-consumption, resulting in salt accumulation if feeding the bioreactor with NO_3_^−^ salt. We avoid this with our novel pH–stat approach, where the reactor is supplied with 5 M HNO_3_ to compensate for the alkalization, thus sustaining NO_3_^−^-concentration at a level determined by the pH setpoint. Here we present the first feasibility study of this method, growing the model strain *Paracoccus denitrificans* anaerobically to high densities with glucose as the sole C-source and NO_3_^−^ as the N-source and electron acceptor.

**Results:**

Our fed-batch culture reached 20 g cell dry weight L^−1^, albeit with slower growth rates than observed in low cell density batch cultures. We explored reasons for slow growth, and the measured trace element uptake indicates it is not a limiting factor. Bioassays with spent medium excluded accumulation of inhibitory compounds at high cell density as the reason for the slow growth. The most plausible reason is that high metabolic activity led to CO_2_/H_2_CO_3_ accumulation, thus suppressing pH, leading to a paucity in HNO_3_-feeding until N_2_-sparging had removed sufficient CO_2_. The three free intermediates in the denitrification pathway (NO_3_^−^ → NO_2_^−^ → NO → N_2_O → N_2_) can all reach toxic concentrations if the electron flow is unbalanced, and this did occur if cells were glucose-limited. On the other hand, accumulation of polyhydroxyalkanoates occurred if the cells were NO_3_^−^-limited. Carefully balancing glucose provision according to the HNO_3_ injected is thus crucial.

**Conclusions:**

This work provides a proof of concept, while also identifying CO_2_/H_2_CO_3_ accumulation as a hurdle that must be overcome for further development and optimization of the method.

**Supplementary Information:**

The online version contains supplementary material available at 10.1186/s12934-024-02595-8.

## Background

The world population is expected to reach 9.7 billion by 2050 [1], and combined with an increasing demand for resource-intensive foods per capita, it is estimated that the production of animal-derived protein must be more than doubled [2]. Such escalation of food production would have grave environmental impacts if provided by conventional agriculture in terms of habitat destruction, resource use, and pollution. This has spurred an interest in single-cell protein (SCP) as an alternative source of protein. Derived from microbial sources [3], SCP emerges as a promising avenue for sustainable food production as it has lower land- and water requirements and greenhouse gas emissions compared to conventional agriculture [4], and allows recycling of waste products [5]. The opportunities seem endless as a wide range of organisms can be cultivated using a variety of substrates and processes [6]. Suitable organisms are selected based on tractability, high growth rates, protein content, and the ability to utilize low-cost substrates or accumulate desirable byproducts. Fungi, algae, and bacteria are most commonly used, and of these, bacteria are attractive because they have the highest protein content (50–80% of dry weight) and are rich in essential amino acids [7].

Submerged high cell density cultivations (HCDC) are most commonly used for SCP production as they maximize volumetric productivity while reducing operating volume, water consumption, and production cost [8]. Biomass concentrations of more than 100 g dw L^−1^ are reported [9]. However, several considerations must be addressed to operate an HCDC for SCP production. The total nutrient requirements often surpass toxic levels if supplied instantaneously, necessitating gradual nutrient provision [10]. This is further complicated by unbalanced provision of nutrients potentially resulting in by-products such as polyhydroxyalkanoates (PHAs) [11]. Another challenge is that conventional HCDC is based on aerobic respiration, and due to the low solubility of O_2_ in liquid, the provision of O_2_ becomes the rate-limiting step [12]. Hypoxia due to inadequate provision of O_2_ may lead to fermentation and accumulation of undesirable byproducts. Despite this, the potential of alternative electron acceptors remains largely untapped. Among these, nitrate (NO_3_^−^) emerges as a promising prospect due to its high solubility in water, and the favorable thermodynamics of its reduction to nitrogen gas (N_2_) via denitrification, i.e. anaerobic respiration sustained by stepwise reduction of NO_3_^–^ to N_2_ via nitrite (NO_2_^−^), nitric oxide (NO) and nitrous oxide (N_2_O) [13]. Denitrifiers are a diverse group of microorganisms, typically facultative anaerobes and non-fermenting, that can utilize a wide range of substrates and metabolic strategies, including hydrogenotrophic denitrification where biomass is produced from molecular hydrogen (H_2_) and carbon dioxide (CO_2_) [14]. This metabolic versatility and the widespread distribution of denitrifiers further warrant investigations of their potential for HCDC and future SCP production.

The standard free energy change (∆G^0^) per electron for denitrification is nearly equal (~ 95%) to that for aerobic respiration [15]. This means that the growth yield (biomass per mole of substrate) for denitrification could nearly equal that of aerobic respiration. This is not the case for canonical denitrifying organisms, however, as their anaerobic respiratory pathway generates 30–40% less proton motive force (*pmf*) per electron than the aerobic counterpart [16]. Organisms with higher *pmf*-generation per electron for N_2_O-reduction have been found [17, 18], and we foresee the discovery of organisms with a higher *pmf*-yield for the other steps of denitrification, thus with anaerobic growth yields nearing those observed under aerobic conditions. Emulsion culturing, selective for high growth yield [19], could assist in finding such organisms.

The solubility of NO_3_^−^ in water facilitates its provision at high rates, which is crucial for efficient production of SCP. However, denitrification-based HCDC is far from trivial: NO_2_^−^, NO, and N_2_O are free intermediates in denitrification, and they are all toxic in various ways. Nitrous acid (HNO_2_) has extensive toxicity, either by dissipation of *pmf,* inhibition of the *pmf*-generation, or both [20], NO and HNO_2_ both nitrosylate iron-sulfur proteins [21], and N_2_O oxidizes cobalt, thus inhibiting corrinoid-dependent pathways (Sun et al. [22], and references therein). Although the regulatory network of denitrification in most organisms secures homeostatic low concentrations of intermediates [23], rampant accumulation of NO to inhibitory concentrations can occur [24, 25]. Another challenge in using denitrification for HCDC is pH-control and salt accumulation. Reduction of NO_3_^−^ leads to alkalization, so the pH must be regulated by adding acid, which is not trivial at high cell densities. If providing NO_3_^−^ as a salt, salt accumulation is inevitable, eventually reaching molar, hence inhibitory, concentrations. Overcoming these challenges holds the key to unlocking vast possibilities within anaerobic HCDC, paving the way for innovative applications in microbial cultivation.

**Fig. 1:**
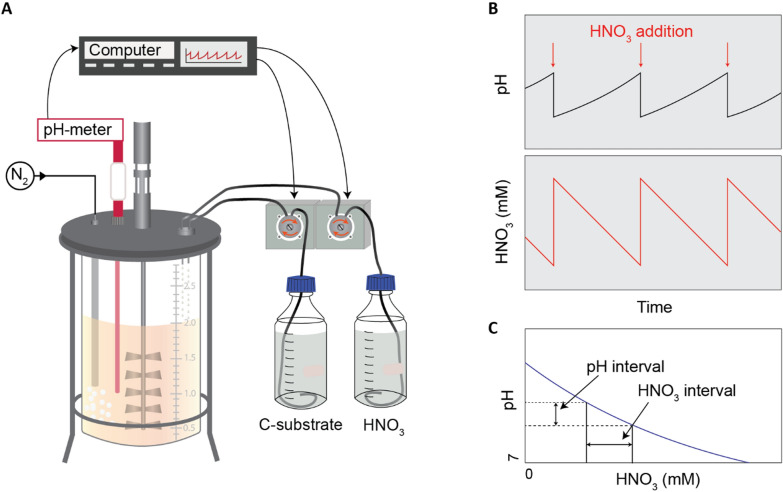
Principle of anaerobic high cell density culturing (HCDC) as fed-batch. **A** The culture is fed by peristaltic pumping of HNO_3_- and glucose-solutions, and by manual injection of trace element solutions. The feed rate is controlled by measured pH, ensuring that pH is kept within a narrow interval. The culture is sparged with N_2_ to remove CO_2_. **B** The respiratory reduction of HNO_3_ to N_2_ raises the pH, which is compensated by pumping a dose of HNO_3_. **C** Based on the titration curve of the growth medium, the pH interval translates into an HNO_3_ concentration interval. Our results indicated that the relationship between pH and HNO_3_ concentration could be obscured by high CO_2_ concentrations (lowering the pH), which would require a refined control algorithm taking monitored CO_2_ concentrations into account

We initiated the development of a robust HCDC process based on denitrification, intending to tackle challenges and circumvent pitfalls. Here we demonstrate that the solution lies in a simple yet effective maneuver—providing NO_3_^−^ as nitric acid (HNO_3_), as described in our patent application [26]: by maintaining pH within a narrow setpoint range, sustained availability of NO_3_^−^ is secured without adding NO_3_^−^ salts (Fig. 1). The alkalization resulting from denitrification is monitored by a pH sensor, initiating the addition of HNO_3_, which subsequently triggers a proportional addition of the carbon feed according to the substrate/biomass stoichiometry. The provision of mineral elements (K, P, S, Mg, Fe, Ca, Mn, Zn, Cu, Mo, Co, Ni) should match the uptake by the cells, to avoid limitation or accumulation to toxic levels.

Using this method, we were able to grow the model denitrifier *Paracoccus denitrificans* on a single C-source (glucose) to a cell density of more than 20 g dry weight L^−1^ in an anaerobic fed-batch. These cell densities are orders of magnitude higher than the typical stationary phase in anoxic batch experiments [27]. The initial phases of development also revealed issues to be addressed when moving forward with this process. Although cultures reached high density with the expected biomass yields, the observed growth rate fell below expectation. Follow-up experiments ruled out accumulation of toxic compounds and did not indicate mineral limitations. Instead, the retarded growth was likely due to high partial pressure of CO_2_ significantly suppressing pH, causing a delayed provision of HNO_3_ and carbon substrate. We also observed PHA accumulation, tentatively ascribed to excess glucose relative to the provision of HNO_3_. Despite these challenges, the results are promising, showcasing the potential for anaerobic HCDC.

## Materials and methods

While all HCDC were fed-batch cultivations in a bioreactor (Fig. 1), we ran several bioassays in small vials (120 mL serum vials) which were monitored for gas kinetics (NO, N_2_O, N_2_, CO_2_) and cell density. The bioassays were conducted at low densities and provided basic parameters for unrestricted anaerobic growth, which were needed for designing the fed-batches. They were also used to check the status of the cells in the fed-batches (inoculating cells from the HCDC in small vials with fresh basal medium) and to test if the HCDC accumulated growth-restricting compounds (fresh cells from small-density cultures inoculated in spent fed-batch medium).

### Strains, media, and culturing conditions

#### Strains

All experiments were carried out using the model denitrifier *P. denitrificans* Pd1222. For some of the experiments, we used a modified Pd1222 strain, where an *mCherry-nirS* fusion gene replaces the native *nirS* gene [28], allowing tracking of the NirS expression in single cells by fluorescence microscopy. The mutant has been validated for anaerobic cultivations and has a practically equal phenotype to the parent strain [28].

#### Media and culturing methods

Cultivations were carried out in a mineral base medium based on Hahnke et al. [29] containing 0.5 g L^−1^ MgSO_4_ · 7H_2_O, 0.1 g L^−1^ CaCl_2_ · 2H_2_O, 1.97 g L^−1^ KH_2_PO_4_ and 14.9 g L^−1^ K_2_HPO_4_, or a modified basal mineral medium, where the concentrations of KH_2_PO_4_ and K_2_HPO_4_ were reduced to 0.99 and 7.45 g L^−1^, respectively. To provide trace elements, we explored different trace element compositions (TE-1 to 3, Table 1) and designed trace element solutions that were added manually in the fed-batch experiments (TRES-2 and 3, Table 1). The trace element compositions and solutions evolved as we gained experience with the cellular uptake of elements in the experiments. All cultivations were done at 30°C at pH 7–7.5.

**Table 1:** Trace element concentrations in the basal mineral medium and the trace element solutions injected during fed-batch

		EDTA	ZnSO_4_ · 7H_2_O	FeSO_4_ · 7H_2_O	MnSO_4_ · H_2_O	CuSO_4_ · 5H_2_O	Co(NO_3_)_2_ · 6H_2_O	H_3_BO_3_	Na_2_MoO_4_· 2H_2_O	NiCl_2_ · 6H_2_O
Concentration in basal mineral media (µM)	TE-1	6.0	38.0	18.0	9.1	1.6	0.85	1.8	1.0	
	TE-2	5.3	38.0	18.0	9.1	1.6	1.0	1.8	1.0	
	TE-3	5.2	38.0	3.6	9.1	1.6	1.0	1.8	1.0	
Concentration in solutions injected in fed-batch (mM)	TRES-2		4.5	200.0	33.0	15.0	0.93	0.3	1.9	0.02
	TRES-3	60.0	380.0	180.0	91.0	16.0	8.5	18.0		

The upper part of the table shows the initial concentration (µM) of trace elements in the basal media for the different experiments. TE-1 was the initial trace element concentration in the M1 and M2 medium for the determination of growth parameters (Table 2), in Fed-batch 1 and in the batch experiment for testing the reservoir solutions. In addition, TE-1 was the concentration of the trace elements in the HNO_3_ solution in the reservoir used for Fed-batch 1. TE-2 and TE-3 were the initial concentrations in Fed-batch 2 and 3 (and associated batch experiments), respectively. The lower part shows the concentrations (mM) in the concentrated trace element solutions designed for the fed-batch cultivation. TRES-2 (0.2 mL L^−1^) was added to Fed-batch 2 and TRES-3 (0.1 mL L^−1^) to Fed-batch 3.

The strains were raised from frozen glycerol stocks under aerobic conditions in 50 mL basal mineral medium supplemented with 20 mM glucose and 5 mM KNO_3_ in 120 mL serum flasks stirred at ≥ 600 rpm using triangular magnetic stirring bars. Cells were adapted to anoxia by transfer to new crimp sealed serum vials with the same medium and He in the headspace (repeated cycles of evacuation and He-filling) with 1 vol% O_2_ (injected after He flushing).

### Chemical analyses

#### Nitrite and nitrate measurements

Liquid samples from the cultures were centrifuged (10,000 ×*g*, 5 min) to remove cells, and 10 µL of the supernatants were injected into a purge vessel containing reducing agents to convert NO_3_^−^ + NO_2_^−^ or only NO_2_^−^ to NO, depending on the reducing agent [30]. The reducing agent was saturated vanadium chloride (VCl_3_) in 1 M HCl (maintained at 95°C) for NO_3_^−^ + NO_2_^−^ or 1% w/v sodium iodide (NaI) in acetic acid (room temperature) for NO_2_^−^. N_2_ was bubbled through the system to maintain anoxic conditions and to transport the generated NO to a chemiluminescence detector (Nitric Oxide Analyzer NOA 280i, General Electric).

#### Glucose measurements

During fed-batch experiments, glucose was measured using an adaption of the method for monitoring blood glucose to get a rapid estimation of the concentration in the reactor. 4 µl of fresh blood was mixed with 1 µl of supernatant before application to a Contour^®^ XT device (Ascensia Diabetes Care Holdings AG). MQ water and 20 µM glucose were used as controls. Selected samples were also stored at –20°C awaiting HPLC analysis for more accurate quantification.

#### Cell density measurements

Cell density was assessed by measuring OD_660_ with MQ-water serving as a blank. Samples were diluted with MQ-water to ensure measurements fell within the linear range of the spectrophotometer (OD_660_ < 0.8). During harvest for dry weight determination, the cell suspensions were pelleted by centrifugation (10,000 ×*g*, 5 min). The pellets were washed twice in MQ water before drying at 100°C until constant weight.

#### Polyhydroxyalkanoate (PHA) detection

The presence and size of PHA granules in the cells were inspected by fluorescence microscopy of cells stained by Nile red [31]: 40 µL of Nile red solution (80 µg/ml dissolved in DMSO) was added to 100 µL of cell suspension that had been washed in MQ water and resuspended in 1 mL water. The mix was incubated for 30 min at room temperature. Then, the cell pellet was washed three times by centrifugation and water resuspension cycles (10,000 ×*g*, 5 min), and inspected for PHA granules by fluorescence microscopy.

#### Fluorescence microscopy

The microscope used was a Zeiss AxioObserver connected to an ORCA-Flash4.0 V2 Digital CMOS camera. A 100 × phase-contrast objective was used and the images were visualized with the ZEN Blue software. We used an HXP 120 Illuminator as the fluorescent light source for detection and quantification of Nile red (636 nm, 390 ms exposure) and mCherry (610 nm, 1000 ms exposure). The images were processed in ImageJ v1.54. Fluorescence intensity in each cell was determined using the MicrobeJ plug-in [32].

#### Inductively coupled plasma mass spectrometry (ICP-MS)

The concentrations of trace metals were determined for liquid medium samples and in cells. All liquid samples were acidified with Ultrapure (UP) HNO_3_ and diluted before analysis. The cell samples were digested in 0.75 mL UP HNO_3_ and left at 100°C until dry. After drying, cell samples were redissolved in 10% (v/v) HNO_3_ and further diluted depending on initial amounts of biomass. The samples were analyzed on a triple quadrupole ICP-MS (Agilent 8900 #100) in He-KED, Oxygen- and Ammonia reaction mode. An external calibration, from certified standards, was used to quantify the elements. No certified reference material was used due to a lack of suitable CRM. Instead, an in-house control standard (1643H; equal to CRM NIST 1643e) was used for checking the calibration for random and systematic errors. The limit of detection (LOD) and limit of quantification were calculated from 3 and 10xSD of blank Eppendorf tubes digested following the same procedure as the cell samples.

#### Headspace gas chromatography (HS-GC)

The concentrations of volatile compounds (acetaldehyde, ethanol, acetone, diacetyl, 2-butanol, and acetoin) were determined in the spent medium by HSGC according to the method described by Grønnevik et al. [33]. Briefly, samples harvested during HCDC were centrifuged (10,000 ×*g*, 10 min) before filtration (Filtropur S 0.2 µm, Sarstedt) and storage at –20°C awaiting analysis. The analysis of samples and standards was conducted as detailed in Dysvik et al. [34] using a 7679A automatic headspace sampler connected to a 6890 GC system with a flame ionization detector (Agilent Technologies) controlled by the Open Lab CDS software (Version 2.7, Agilent Technologies).

#### High-performance liquid chromatography (HPLC)

The concentrations of glucose and organic acids (α-ketoglutaric acid, pyruvic acid, lactic acid, formic acid, and acetic acid) in the spent medium were determined by HPLC as described by Grønnevik et al. [33]. Cell-free samples were filtered using a 0.2 µm PTFE membrane (Acrodisc CR 13 mm Syringe filter, PALL, GB) and transferred to HPLC vials before analysis using the HPLC-instrument Agilent Technologies 1260 Infinity II (Agilent Technologies, Singapore) equipped with an Aminex HPX-87H column (Bio-Rad Laboratories, Hercules, CA), a diode array detector-ultraviolet (DAD-UV) detector for quantification of organic acids and a Refractive Index (RI) detector for quantification of glucose and acetic acid.

### Small-batch experiments (Bioassays 1–4)

Batch cultivation in 120 mL serum vials was used to assess the physiological parameters needed for designing the fed-batch process (growth rates, growth yield, gas kinetics, and trace element uptake). This format was also used in bioassays during high cell density culturing in fed batches, designed to assess the physiological condition of the cells, and whether the low growth rates at high cell density could be due to accumulation of inhibiting compounds.

The vials were placed in an incubation robot designed for monitoring gas kinetics in up to 30 parallel stirred cultures. The robot is described in detail by Molstad et al. [35], and the refined version used in our experiments is described by Molstad et al. [36]. In brief: small-batch experiments were conducted in 120 mL serum vials containing 50 mL medium, a Teflon coated magnetic stirring bar, with He (or He + O_2_) in the headspace (to enable quantification of N_2_ production). The vials were placed on submersible magnetic stirring plates in the thermostatic water bath (30°C) of the incubation robot which samples the headspace of each vial at intervals (minutes to hours depending on the experiment) for measuring O_2_, CO_2_, N_2_O, and N_2_ by gas chromatography (Agilent GC − 7890A) and NO by chemiluminescence (Model 200A, Advanced Pollution Instrumentation, San Diego, USA). After each sampling, an equal volume of He is returned to the headspace, to maintain ~ 1 atm pressure and minimize leakage of air into the vial. Elaborate routines are used to calculate rates of production or consumption for each gas, taking solubility, gas transport coefficient, leakage, and sampling loss into account [37]. This allows estimations of apparent specific growth rates (µ, h^−1^) based on nonlinear regression of total electron flow (mol e^−^ vial^−1^ h^−1^) to terminal electron acceptors, and biomass yield per mol e^−^ for aerobic and anaerobic growth. The system has been used in numerous physiological studies of denitrifying bacteria and communities [25, 27, 28, 38, 39].

### Fed-batch experiments (Fed-batches 1–3)

In our first attempt at HCDC (Fed-batch 1), we used a Minifors bench-top bioreactor from Infors HT, with a 2.5 L glass vessel and a working volume of 2 L. In all later fed-batch experiments, we used the more recent model Minifors 2 system with a 1.5 L glass vessel and a working volume of 1 L. The liquid was stirred at 250–500 rpm with one six-flat-blade disc turbine and one propeller. The temperature was set to 30°C and the outgas cooling was connected to a water bath at 8°C. The reactor was continuously sparged with N_2_ via a submerged sparger at an initial rate of 0.1 L h^−1^. pH was monitored using a Mettler Toledo 405-DPAS-SC-K8S/120 pH Probe. Dissolved O_2_ was monitored using the Hamilton OxyFerm FDA 225 probe. In the Minifors 2 set-up, four peristaltic pumps were connected to the reactor; an acid and base pump controlled by the pH, an antifoam pump controlled by a level sensor, and a feed pump that could be controlled at the user’s discretion. The older Minifors system did not allow for a freely controlled feed pump, hence glucose was injected manually. At regular intervals, liquid samples were taken for OD_660_, NO_3_^−^, NO_2_^−^, and glucose measurements and headspace samples were transferred to 15 mL sealed and evacuated glass vials for NO, N_2_O, and CO_2_ measurements using the robotized incubation system. At more irregular intervals, fed-batch cultured cells and supernatant were sampled and used in small-batch bioassays or stored for further chemical analysis.

## Results and discussion

**Fig. 2:**
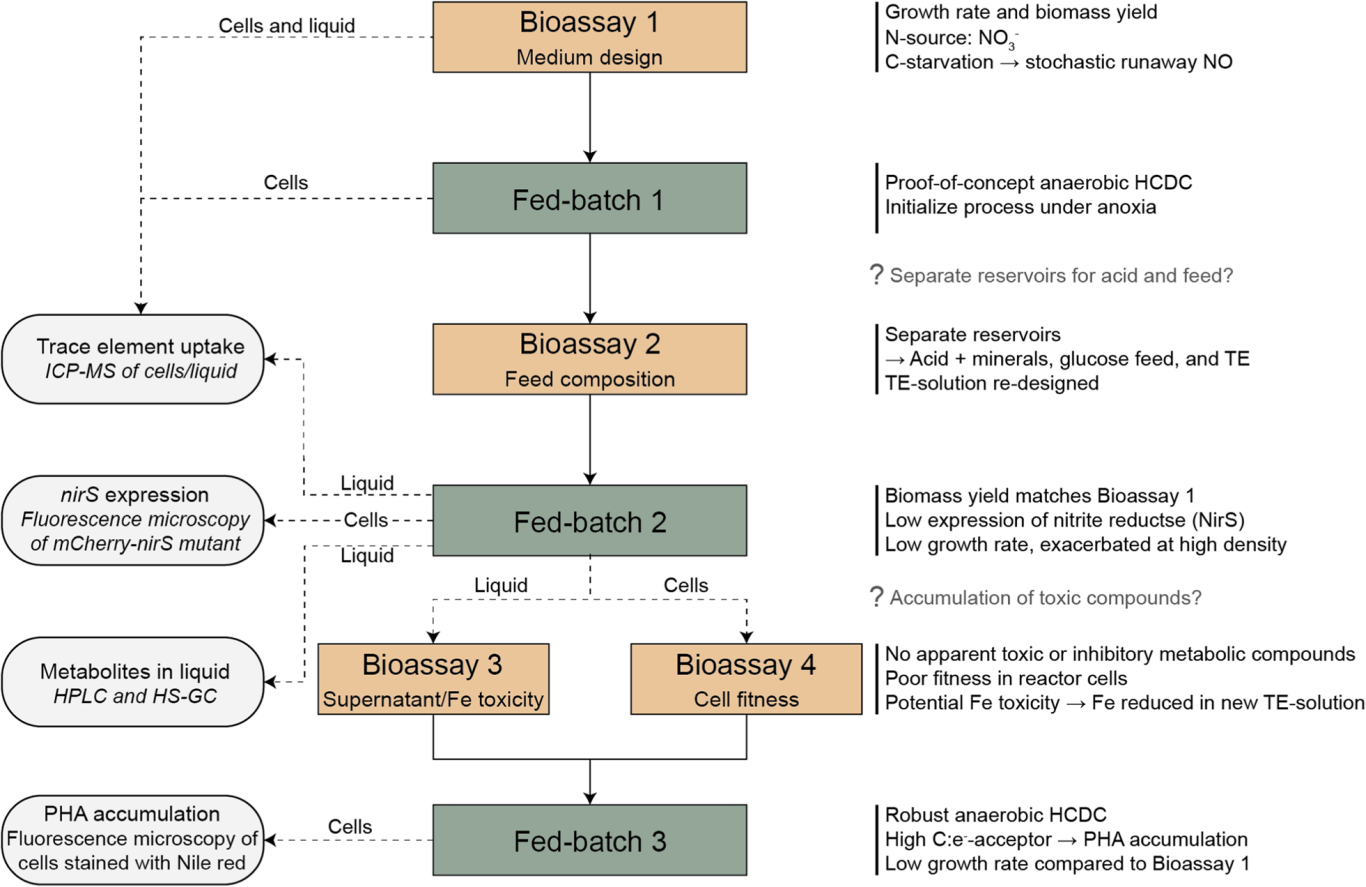
Graphic summary outlining the bioassays and fed-batches. Bioassay 1 was used to determine the growth parameters and yield for anaerobic growth in the designed medium, which was then used in an initial attempt at high cell density cultivation in a fed-batch (Fed-batch 1; Strain: *Pd1222*). There were indications of toxicity when mixing glucose into the acid reservoir. To address this, we designed a bioassay to test the different feed compositions and combinations (Bioassay 2). Based on the results, we concluded that the acid, feed, and trace elements should be provided from three separate reservoirs. Cells and liquid samples from Bioassay 1 and Fed-batch 1 were analyzed by ICP-MS to determine the trace element content, which was used to re-design the trace element solution (TRES-2, Table 1) for Fed-batch 2. Fed-batch 2 (Strain: *Pd1222*_*mC-nirS*) had the expected biomass yield, but the growth rate was lower than expected based on Bioassay 1. During Fed-batch 2, cell-free liquid samples were analyzed by ICP-MS, HPLC, and HS-GC to determine the concentrations of trace metals and selected metabolites. Cells were analyzed by fluorescence microscopy to determine the expression of *mCherry-nirS*. At the end of Fed-batch 2, cells and reactor liquid were separated and used in Bioassay 3 and 4, respectively. Bioassay 3 aimed to test the presence of inhibitory or toxic compounds in the liquid by incubating with fresh cells. In addition, the toxicity of Fe(II) and Fe(III) under anoxic conditions was assessed. Bioassay 4 tested the fitness of the reactor cells by inoculation in a fresh medium. Bioassays 3 and 4, and the results from the liquid analysis, prompted us to re-design the trace element solution prior to Fed-batch 3 by reducing the Fe content. In Fed-batch 3 (Strain: *Pd1222*) we sustained a robust anaerobic HCDC, however, glucose excess resulted in PHA accumulation and the growth rate remained low

The experimental work is outlined in Fig. 2 and details the bioassays and fed-batch experiments.

### Bioassay 1: Determination of growth rate, biomass yield, and trace element uptake

**Fig. 3:**
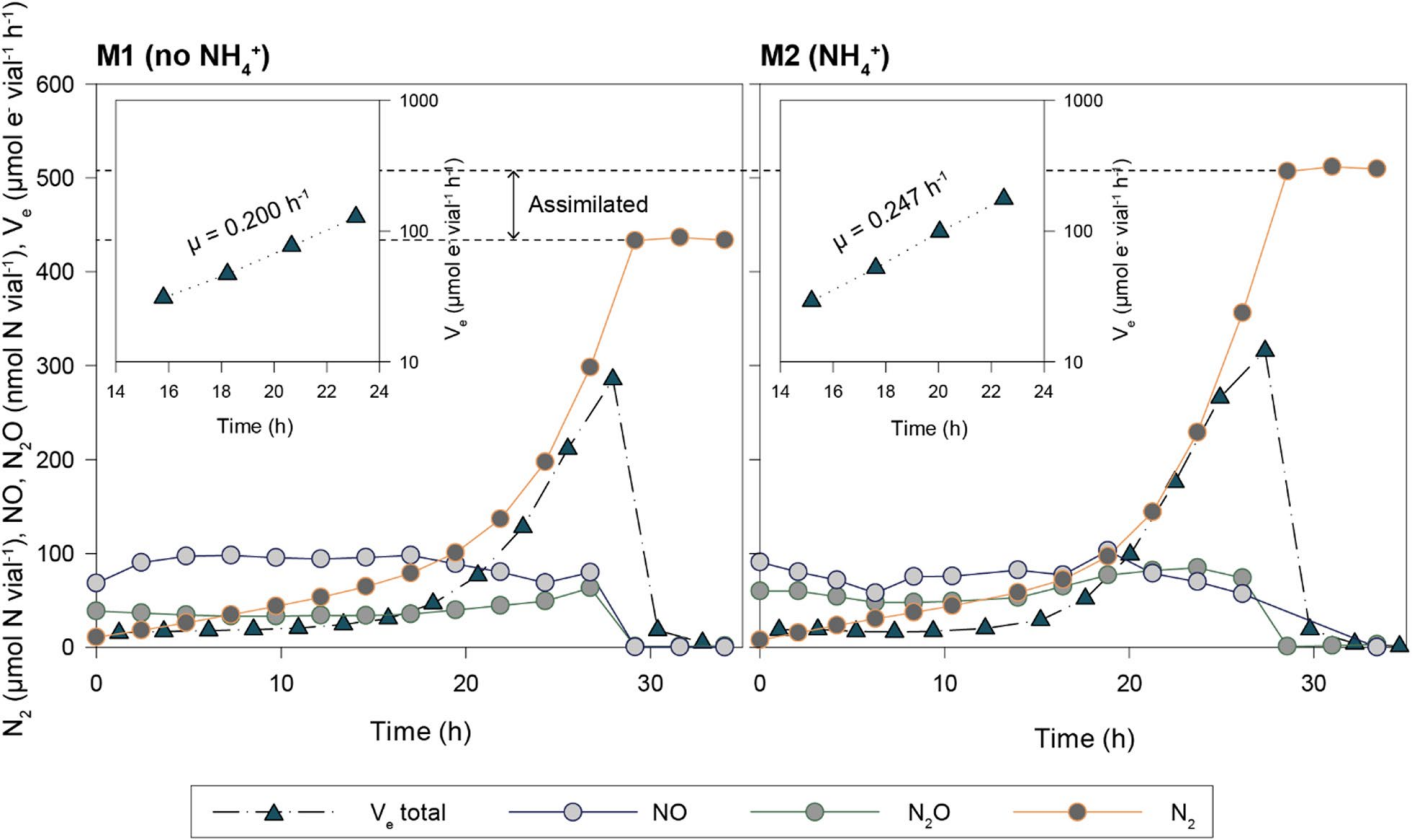
Gas kinetics in *P. denitrificans* growing under NO_3_^−^ limitation, with (M2) and without (M1) NH_4_^+^. This show the results in representative single vials. Main: accumulation of N_2_ (µmol N vial^−1^), NO, and N_2_O (nmol N vial^−1^) during anaerobic growth by denitrification. Inserts: e^−^-flow rates to N-oxides, V_e_ (µmol e^−^ vial^−1^ h^−1^), and apparent specific growth rate (µ, h^−1^) as estimated by nonlinear regression of rates against time

We have previously conducted extensive physiological analyses of *P. denitrificans* during aerobic and anaerobic growth in Sistrom’s medium with succinate as the carbon source and ammonium (NH_4_^+^) as the nitrogen source [24, 27, 40, 41]. Although defined and relatively simple, Sistrom’s medium contains a number of redundant components, and for the purpose of anaerobic HCDC, we designed a simpler basal mineral medium based on the work by Hahnke et al. [29]. Moreover, as the consumption of succinic acid would interfere with the pH–stat of the anaerobic HCDC process, we opted for glucose as the sole C-source and wanted to explore the possibility of using NO_3_^−^ as the sole nitrogen source for assimilation. This redesign necessitated a thorough physiological assessment prior to attempting anaerobic HCDC with *P. denitrificans.* A series of small-batch experiments (Bioassay 1, Fig. 3; further details in Additional File A) were conducted to measure growth rate and yield in the basal mineral medium with millimolar concentrations of NO_3_^−^ and glucose. Cultures in Sistrom’s medium containing succinate and NH_4_^+^ as used previously (e.g. Bergaust et al. [27]) were included as a point of reference. The biomass yield per mol glucose and NO_3_^−^ was estimated in glucose and NO_3_^−^ limited cultures, respectively, and the apparent metabolic cost of NO_3_^−^ assimilation vs NH_4_^+^ assimilation was assessed by comparing growth rates and yields in batch cultures with (M2) and without (M1) NH_4_^+^. The results (Table 2) show that forcing the cells to assimilate NO_3_^−^ (M1) instead of NH_4_^+^ (M2) reduced the growth rate and growth yield by ~ 17% and ~ 24%, respectively. The recovery of NO_3_^−^-N as N_2_ indicated that approximately 15% of the NO_3_^−^ consumed in M1 was assimilated (M1 versus M2, Fig. 3).

**Table 2:** Growth rates and -yields in denitrifying batch cultures with glucose in basal mineral medium with- and without NH_4_^+^ as determined in Bioassay 1

Medium	Growth rate (µ, h^−1^)	Y_C_ (mol cell-C mol^−1^ glucose-C)	Y_N_ (mol cell-C mol^−1^ NO_3_ consumed)	% of NO_3_ assimilated	Y_Nden_ (mol cell-C mol^−1^ NO_3_ respired)
M1 (no NH_4_^+^)	0.19 ± 0.02 (n = 6)	0.28 ± 0.01 (n = 7)	0.57 ± 0.01 (n = 7)	15 ± 1	0.49
M2 (NH_4_^+^)	0.23 ± 0.02 (n = 6)	0.37 ± 0.002 (n = 7)	0.75 ± 0.06 (n = 7)	0	0.73

Of note, the anaerobic growth rate in Sistrom’s medium with succinate and NH_4_^+^ was 0.31 h^−1^, which is 35% higher than in M2 (glucose + NH_4_^+^). The reason for the higher growth rate in Sistrom’s medium is plausibly that succinate is a better C-substrate, but could also be due to the presence of glutamic acid (0.1 g L^−1^) and aspartic acid (0.04 g L^−1^) in the Sistrom’s medium.

Anaerobic growth rates (µ h^−1^) were estimated for each vial based on the total e^−^-flow to terminal e^−^-acceptors (V_e_, µmol e^−^ vial^−1^ h^−1^) in cultures provided with a surplus of glucose. Yields per mol N and C (Y_N_ and Y_C_) were estimated based on measured cell dry weight at endpoint (N or C depletion) in cultures limited in NO_3_^−^ and glucose, respectively. Cultures (n = 3) in Sistrom’s medium with succinate as C-source and NH_4_^+^ as N-source were included for comparison, and the estimated anaerobic growth rate in these vials was 0.313 ± 0.005 h^−1^ (not shown in table). Aerobic growth rates in each medium are shown in Figure S1 in Additional File A.

**Fig. 4:**
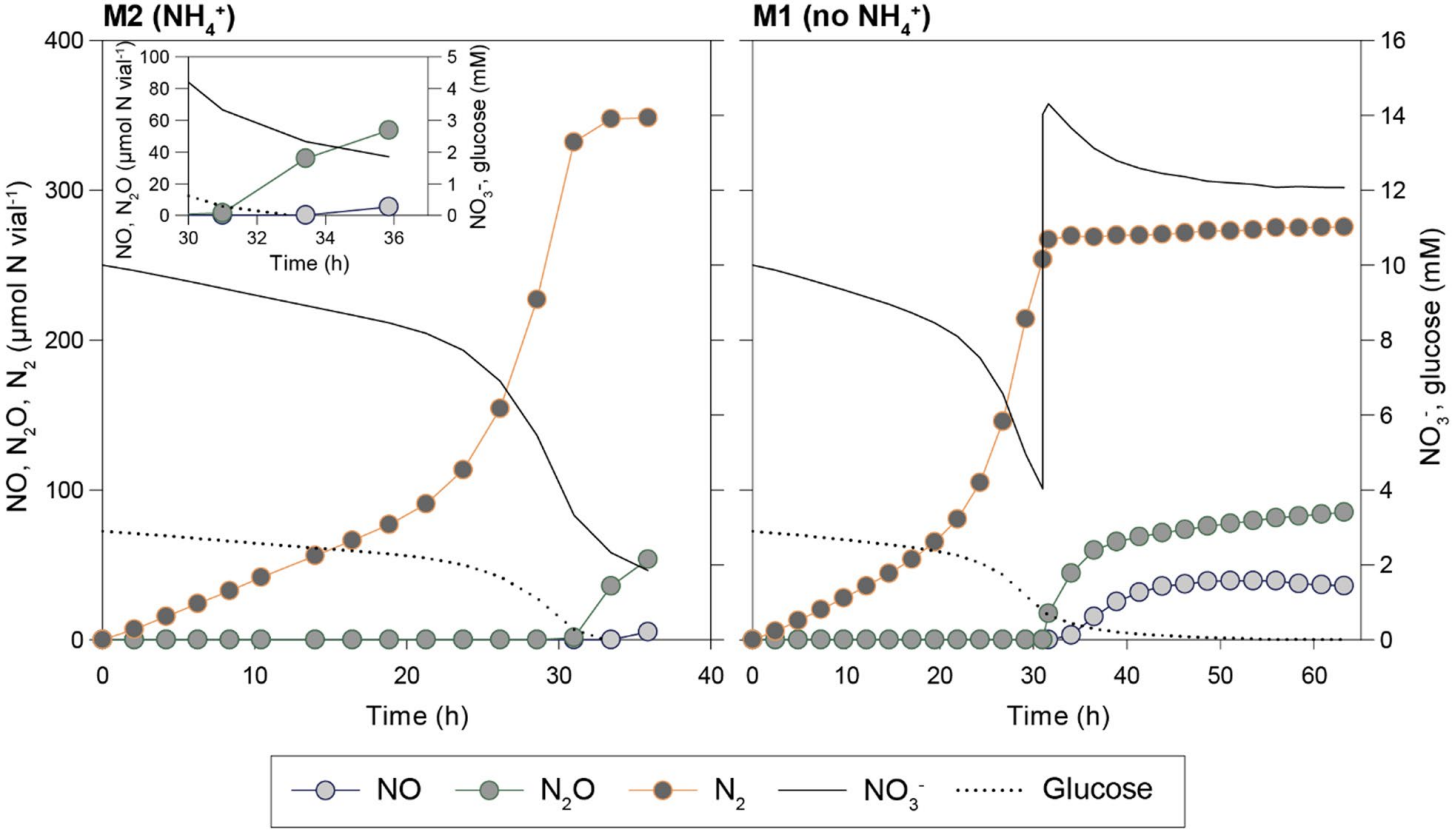
Stochastic accumulation of NO and N_2_O in single vials in response to C-starvation and injection of NO_3_^−^. Runaway accumulation of NO occurred in two out of six glucose-limited cultures (2.9 mM glucose and 10 mM NO_3_^−^) in Bioassay 1. This show measured N_2_, N_2_O, and NO (primary y-axis; µmol N vial^−1^), and the NO_3_^−^ and glucose (secondary y-axis; mM) as estimated based on the measured N-gas production and the stoichiometry of growth (Table 2) in two single vials (M1 and M2)

In most cultures, NO and N_2_O concentrations remained in the nmolar range during the transition to anoxia and subsequent denitrification. This was in line with previous results for *P. denitrificans* which demonstrated a strict control of gaseous N-oxides in anaerobic cultures [23, 27, 42]. However, this robustness is evidently challenged under C-limitation: When faced with C-depletion, a subset of vials, both with- and without NH_4_^+^, accumulated micromolar concentrations of NO and N_2_O (Fig. 4). In M2, NO and N_2_O started to accumulate in response to glucose depletion. In M1, a second dose of KNO_3_ (0.5 mL 100 mM KNO_3_) after 31 h aggravated the NO- and N_2_O-accumulation: NO reached 37 µmol vial^−1^ = 1.28 vol% in the headspace = 23 µM NO in the liquid, plausibly inhibiting Nor (K_i_ for Nor is 13.5 µM, [43]) as well as Nos (K_i_ = 0.35–5.5 kPa [44] = 6–33 µM NO in the liquid). This phenomenon appeared stochastic as it only occurred in two out of six replicates, and suggests that *P. denitrificans* can lose control of NO when C-limited, in stark contrast to its strict homeostatic control of NO when provided with ample amounts of electron donor [23].

The stochastic rampant NO accumulation observed in C-starved cultures illustrates the need for a carefully balanced provision of electron donor and acceptor. The cost of NO_3_^−^ assimilation in terms of growth rate and yield was deemed acceptable, at least during the developmental phase of anaerobic HCDC, and we thus moved forward with the NH_4_^+^- free medium (M1) when assessing trace element requirements and conducting the very first fed-batch culture (Fed-batch 1).

**Fig. 5:**
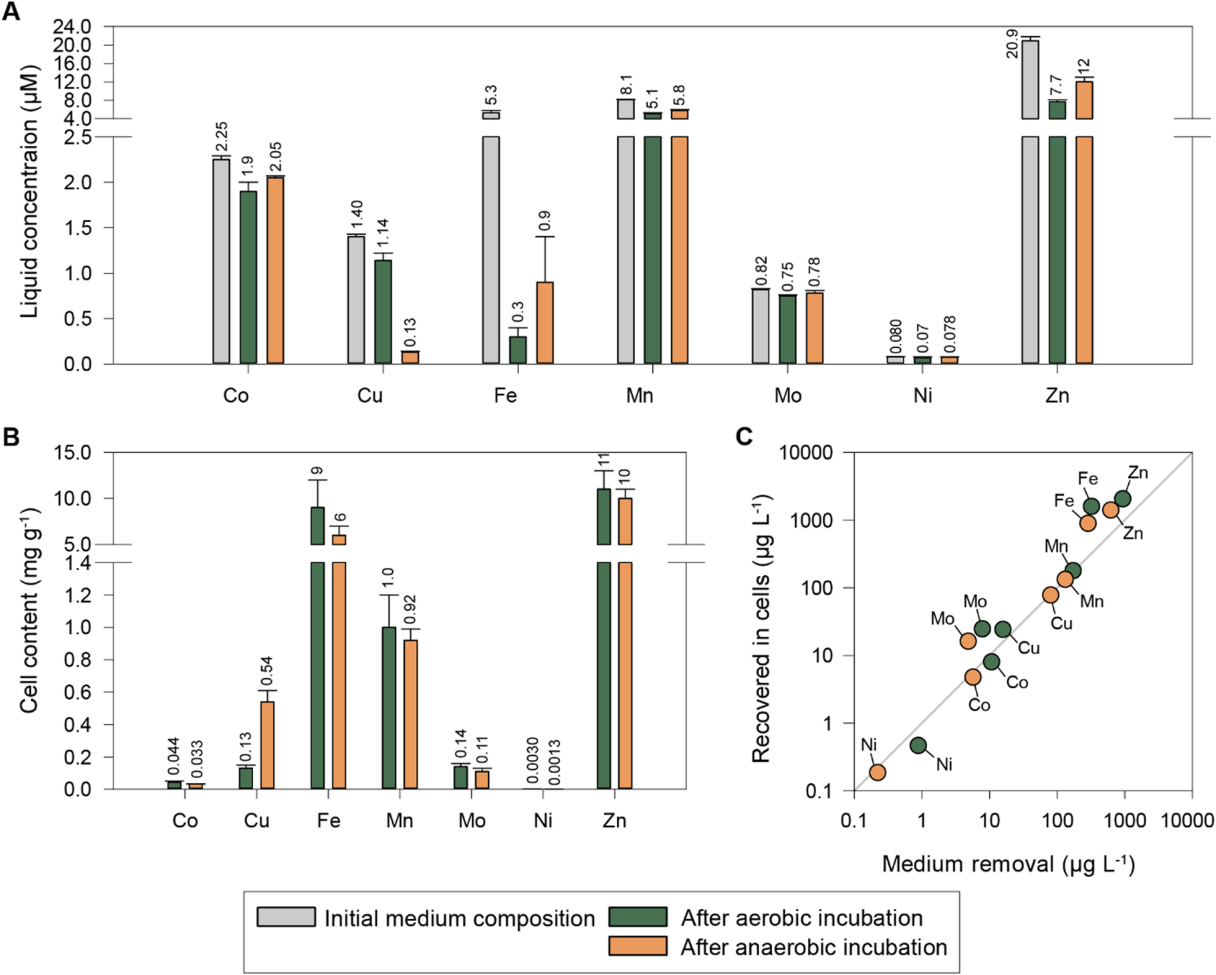
Trace element analysis by ICP-MS after batch cultivation in M1 medium. **A** The initial trace element concentration in the medium and the concentration following aerobic (green) and anaerobic (orange) incubation of *P. denitrificans*. **B** The concentration of trace elements in cells grown aerobically (green) and anaerobically (orange). **C** Comparison of the content of trace elements in the biomass with the calculated uptake from the medium based on trace element concentration in the liquid. The average and standard deviation are based on 3 replicates

In typical batch experiments, where cultures are grown to relatively low densities (< 1 g L^−1^), sufficient trace elements can be secured at low, hence non-toxic concentrations. However, when high density is the aim, the rate of provision and relative concentrations of trace metals must be fine-tuned to secure adequate provision while avoiding toxic concentrations. To design a trace element solution for high cell density cultivation of *P. denitrificans*, we needed data on the element acquisition of the cells during unrestricted growth. ICP-MS was used to measure the residual trace element concentration in the medium (Fig. 5A) and the content in the cells (Fig. 5B) after aerobic and anaerobic growth in batch cultures with M1 medium (Full dataset in Table S1 in Additional File A).

Consistent with the high Cu content of N_2_O reductase, the Cu content of anaerobic cells was more than fourfold higher than in cells grown under oxic conditions. Anoxic conditions also significantly enhanced the uptake of Al, Ba, and Sr, while reducing the uptake of Ni and Zn. The measured contents in the cells were compared to the calculated loss from the medium and the agreement was reasonable except for Mo, Fe, and Zn, where the measured cell content was higher (3.2-, 5.0- and 2.2-fold, respectively) than the calculated loss from the medium (Fig. 5C). The reason for this discrepancy is not known. To secure adequate provision of iron to core enzymes, but at some risk of overdosing, the Fe concentrations in TE-2 and TRES-2 (used in Fed-batch 2) were based on the biomass measurements.

### Fed-batch 1: Anaerobic HCDC is feasible, but challenges arise during the oxic-anoxic transition

Fed-batch 1 (details in Additional File B) was run in a simple bioreactor set-up where the provision of HNO_3_ depended on the pH increase driven by denitrification (pH–stat), but glucose and trace elements had to be injected manually, due to limitations of the bioreactor system used. For this experiment, the primary query was whether pH-dependent anaerobic HCDC with HNO_3_ as e-acceptor and N-source was feasible, and thus the stoichiometry of glucose, macro-, and trace elements was secondary. This simple set-up nevertheless revealed several issues and generated hypotheses regarding the requirements for a successful anaerobic HCDC of *P. denitrificans*. (1) The fed-batch was initiated under oxic conditions with aerobically pre-cultured cells. The transition to anoxia and denitrification was thus driven by aerobic respiration. As conditions became hypoxic, we observed rampant accumulation of NO_2_^−^ (> 20 mM) likely caused by weak initial expression of nitrite reductase (Nir) typical for this strain [28, 45]. We concluded that anaerobic HCDC should be initialized under anoxia with anaerobically adapted cells; (2) Accumulation of CO_2_ may lower the pH in the medium, retarding the pH-dependent provision of HNO_3_, effectively leading to electron acceptor limitation. Sufficient N_2_-sparging and selection of the correct pH-window of operation are thus critical; (3) To simplify the provision of substrates, we tried to mix all ingredients into one reservoir (glucose, macro- and trace-elements, and HNO_3_) at a late stage, but this resulted in growth arrest, plausibly due to generation of toxic compounds (the color of the reservoir changed gradually to deep green); (4) Episodes with exponential growth were obtained, even at moderately high cell density (OD_660_ = 10–20), albeit with ~ 50% lower growth rates than in low cell density batch culture with the same medium; and (5) The trace element content of the cells at the end of Fed-batch 1 was much lower than measured in cells grown in low cell density batch cultures. This could suggest that the trace element requirements are lower than the uptake during exponential growth at low cell density in batch cultures (Fig. 5), but we suspected that it could be due to the overall poor condition of the cells at the time of sampling. On this background, we decided to use the measured uptake in batch cultures (Fig. 5) when designing the trace element solution (TRES-2, Table 1) and the amounts injected during the Fed-batch 2: The relative amounts of copper and iron concentrations were increased (compared to TE-1) and nickel was introduced. The relative amounts of zinc, manganese, cobalt, and molybdenum were reduced as the remaining amount in the spent anaerobic medium exceeded 50% for all. Later measurements of trace element uptake at several stages of Fed-batch 2 showed similar low concentrations in cells as measured at the end of Fed-batch 1 (all measurements are shown in Table 3), indicating that these values should have been used instead of the very high concentrations measured in the batch experiment.

### Bioassay 2: Mixing of reservoir solutions leads to toxicity

It would be practical to combine several feed components in a single feed reservoir, but the first fed-batch experiment indicated that chemical reactions in this mix generated inhibitory compounds, effectively arresting growth. We suspected that reactions between glucose and trace elements were responsible, possibly aggravated by HNO_3_, but could not exclude the generation of inhibitory compounds by reactions with macroelements as well. To explore this, we monitored respiration in denitrifying low cell density batch cultures, in response to injection of various mixtures of 5 M HNO_3_, 3.125 M glucose, macro elements (ME: MgSO_4_, CaCl_2_, K_2_HPO_4,_ and KH_2_PO_4_), and trace elements (TE: ZnSO_4_, FeSO_4_, MnSO_4_, CuSO_4_, Co(NO_3_)_2_, H_3_BO_3_, Na_2_MnO_4_, and NiCl_2_) (Table S2 in Additional File C).

**Fig. 6:**
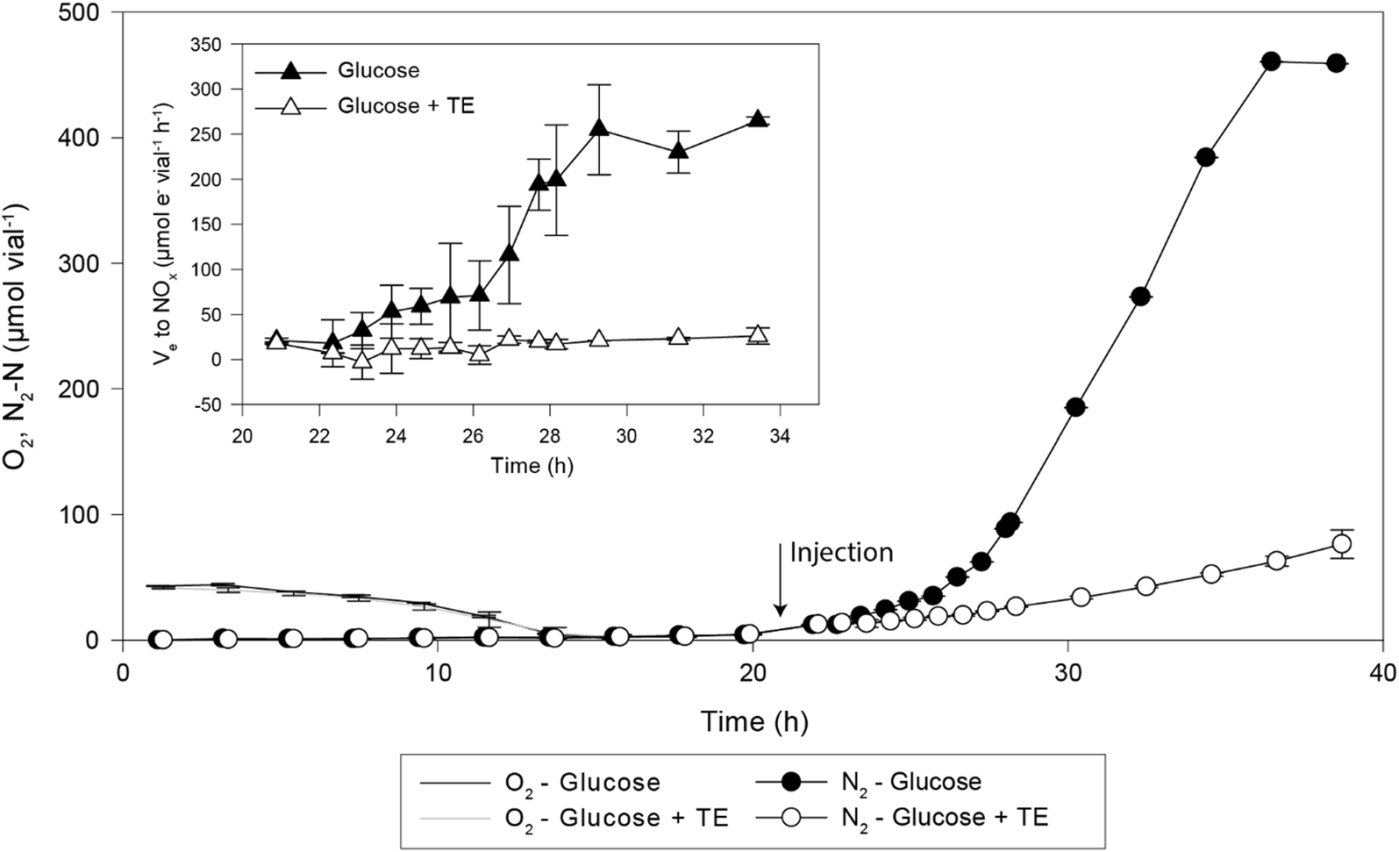
Glucose mixed with trace elements results in growth inhibition. *P. denitrificans* was inoculated in serum vials (50 mL medium M1; initial 1 vol% O_2_ in the headspace, 5 mM KNO_3_, and 10 mM glucose). After depletion of O_2_ and onset of denitrification, all vials got an additional 5 mM KNO_3_ and either glucose solution alone (3.125 M, 0.16 mL vial^−1^) or as a mix with trace elements. The figure shows O_2_-depletion (µmol vial^−1^, line) and subsequent N_2_ production (µmol N vial^−1^, circle) in the cultures (n = 2 replicate vials per treatment, standard deviations shown as vertical bars). The insert shows the estimated electron flow to NOx, V_e_ (µmol e^−^ vial^−1^ h^−1^) in the period after the injection. The result demonstrates that respiration was severely inhibited by the mixture of glucose and trace elements

The results demonstrated an immediate inhibition of growth in the vials that received 60 µL glucose mixed with trace elements (3.125 M glucose and TRES-2, solution left at room temperature for > 1 month prior to use) (Fig. 6); a dose conservatively mimicking an initial injection in fed-batch. The acid mixed with macroelements (ME)**,** and the trace element mixture alone did not appear to result in any inhibitory effect (Figure S3 in Additional File C). Based on these results, we decided to supply substrates from three separate reservoirs: (1) HNO_3_ with macro elements; (2) glucose, and (3) trace elements. The separation of HNO_3_ and glucose was required to facilitate fine-tuning of their relative provision during culturing, and their combination was thus not specifically tested for toxicity.

### Fed-batch 2: Successful high-density cultivation by pH-homeostasis

**Fig. 7:**
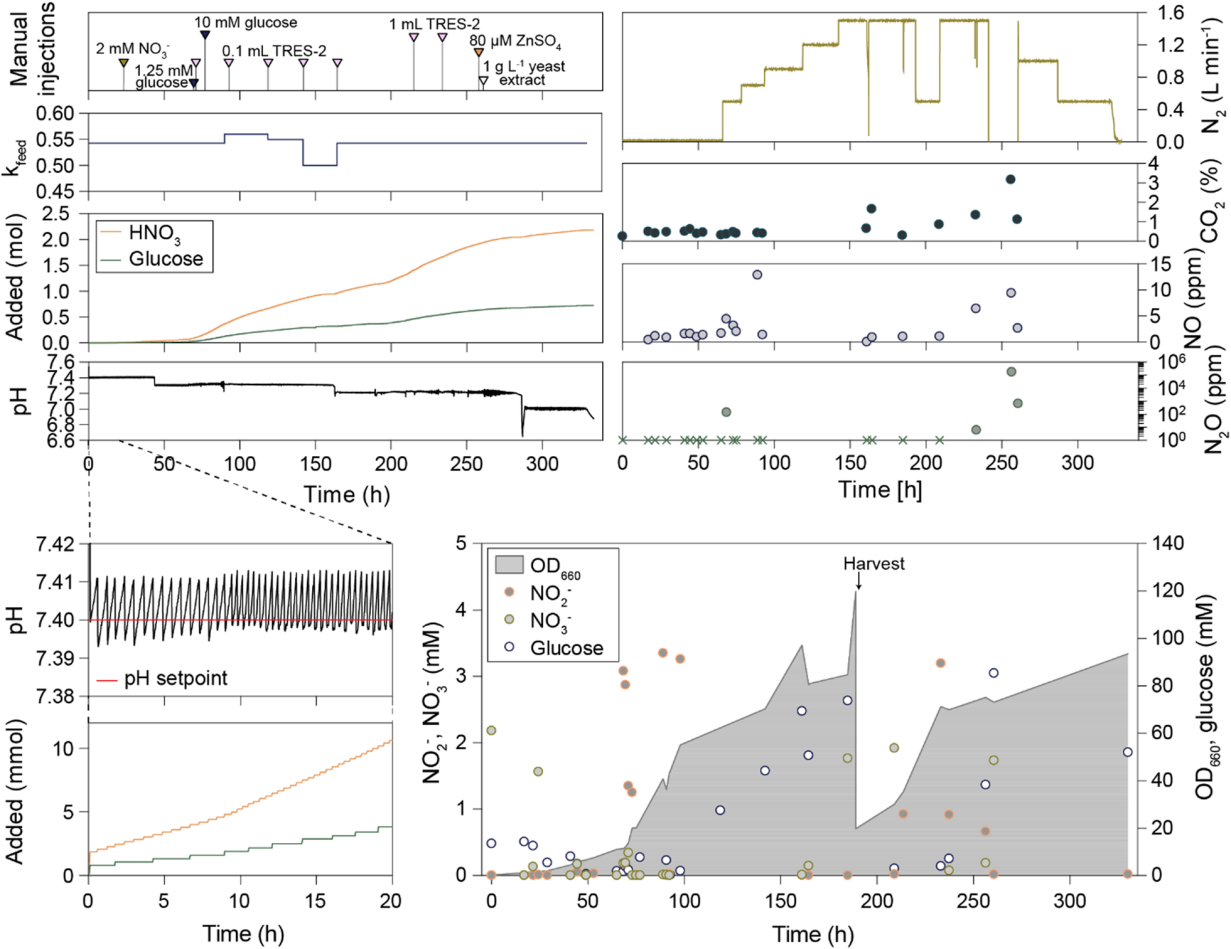
Overview of the 330 h of anaerobic fed-batch cultivation of *P. denitrificans with mCherry-nirS (Fed-batch 2)*. The bioreactor was operated as a pH–stat where denitrification-driven increase in pH (left number four and insert) was monitored and used to initiate the acid feed pump. The *k*_*feed*_ parameter (ratio between glucose- and HNO_3_-feed volume) was adjusted several times to avoid NO_3_^−^-limitations and glucose excess. In addition, substrates were injected manually several times throughout the run, to explore limitations. The manual injections are shown in the top left. The N_2_ sparging flow rate was adjusted in response to increased cell density to limit CO_2_ accumulation in the reactor (upper right). Headspace samples were taken at intervals, and measured for NO, N_2_O, and CO_2_, with results shown in the three mid-right. N_2_O was below the detection limit (2 ppmv) in most samples (marked with a cross on the x-axis). The stirring speed (not shown) was increased from 250 to 350 rpm after 20 h and further increased to 500 rpm after 165 h. The lower right shows the offline measurements of NO_2_^−^, NO_3_^−^, glucose, and OD_660_. The lower left show pH and the cumulated input of HNO_3_ and glucose (mmol) during the first 20 h

In Fed-batch 2, the bioreactor was initialized with 500 mL sterile modified mineral medium (with TE-2) containing 10 mM glucose. The reactor was sparged with N_2_ to remove O_2_ before inoculation with anaerobically raised *P. denitrificans* carrying the *mCherry-nirS* fusion gene*.* Throughout the process, pH was monitored continuously and maintained within narrow limits around a set point by injection of 5 M HNO_3_ containing macroelements (3.15 g L^−1^ MgSO_4_ · 7H_2_O, 0.77 g L^−1^ CaCl_2_ · 2H_2_O, 1.21 g L^−1^ K_2_HPO_4,_ and 1.55 g L^−1^ NaH_2_PO_4_) in response to pH increase due to HNO_3_-consumption. The rate of HNO_3_ provision controlled the pumping of the 3.125 M glucose solution to a set ratio (parameter *k*_*feed*_ = mL glucose solution mL^−1^ acid), while TRES-2 was injected manually at intervals. The reactor was run for 330 h in two consecutive batch cycles of 188 and 142 h, with a maximum observed OD_660_ > 90 for both (Fig. 7).

In the first cycle, the culture grew exponentially to high density as seen by the increase in OD_660_ to ~ 60 during the first 90 h. Unlike in the initially oxic Fed-batch 1, NO_2_^−^ was kept in the low mM range showing that *P. denitrificans* was sufficiently adapted to anaerobic respiration (Fig. 7). The glucose, and NO_3_^−^ concentrations gradually decreased despite their pH-guided provision. To prevent substrate limitation, additional KNO_3_ and glucose were injected manually on two and one occasions, respectively, during the first 75 h. After 90 h the k_feed_ (Fig. 7**,** upper left), was adjusted from 0.543 to 0.560 to increase the provision of glucose relative to HNO_3_. However, instead of spurring an increase in growth rate, the culture grew at a near-linear rate and glucose gradually increased during the next ~ 100 h to a maximum of approximately 80 mM in the reactor toward the end of the first batch cycle (Fig. 7, bottom right panel). A second cycle was initiated after 188 h by harvesting ~ 90% of the culture volume and then filling up to 60% of the original culture volume with fresh basal medium. Again, the culture grew exponentially to high density, but after reaching OD_660_ ~ 80, the growth rate declined to a pace comparable to that seen in the late phase of cycle 1. Several adjustments were attempted to increase the growth rate, including adding additional trace elements, glucose, and NO_3_^−^, zinc (80 µM ZnSO_4_), yeast extract (1 g L^−1^) in case there was any hidden auxotrophy, and increasing the stirring speed. None of these interventions enhanced the growth.

Headspace gases were measured offline in manually retrieved samples by single vial analysis in the robotized incubation system [36]. With few exceptions NO- and N_2_O-concentrations were below 10 and 2 ppmv, respectively (if equilibrium between liquid and headspace, 1 ppmv N_2_O and NO are equivalent to 21 and 1.8 nM in the liquid, respectively). These low NO and N_2_O concentrations reflect a balanced denitrification process during the periods of exponential growth. CO_2_ was produced at a rate dependent on the activity of the culture, increasing with density. To counteract excessive CO_2_ accumulation in the medium, the N_2_-sparging rate was increased several times during the process and the CO_2_ concentration observed in the headspace was kept < 1% during cycle 1 and < 4% during cycle 2 (Fig. 7, upper right).

**Fig. 8:**
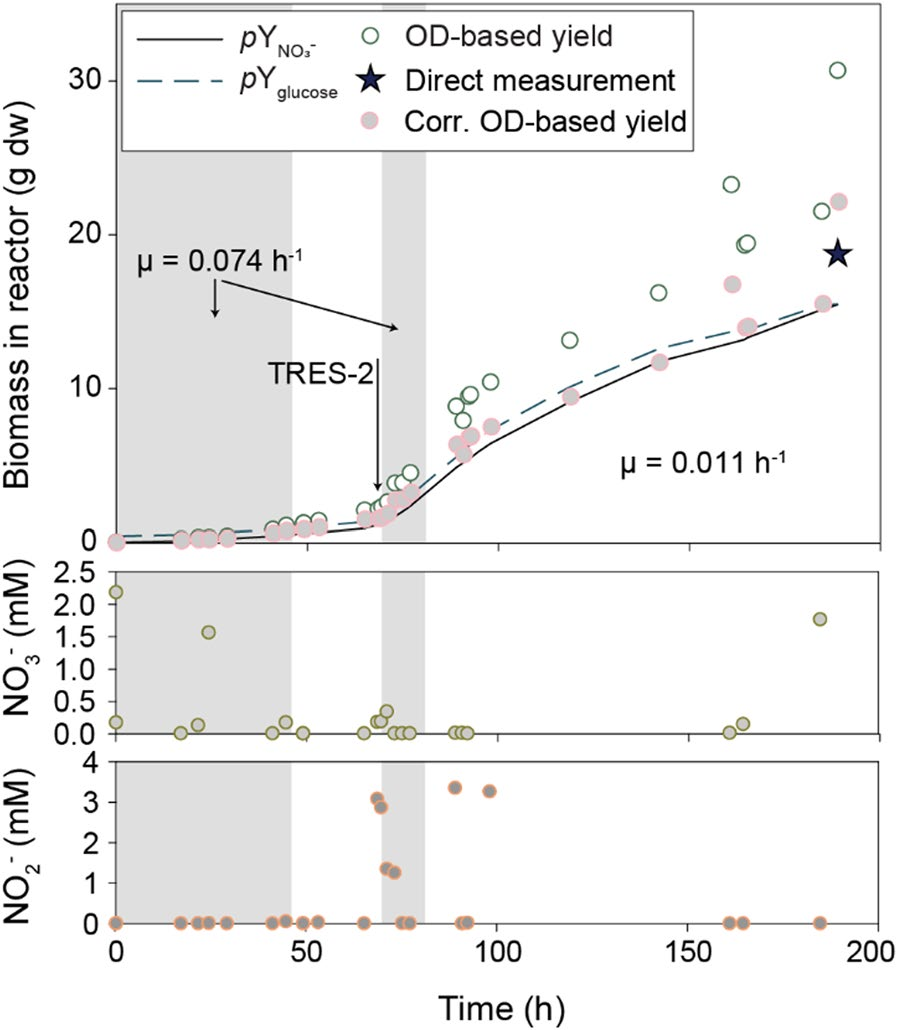
Substrate consumption and biomass production during cycle 1 of Fed-batch 2. The top shows biomass calculated from cumulated consumption of NO_3_^−^ ($\text{pY}_{\text{NO}_3^-}$, assuming $\text{Y}_{\text{NO}_3^-}$ = 13.68 g dw mol^−1^ NO_3_^−^) and glucose (pY_gluc_, assuming Y_gluc_ = 40.32 g dw mol^−1^ glucose), compared with biomass based on OD_660_ during a 190 h fed-batch cultivation. Open circles are the estimated dw based on measured OD, assuming a conversion factor of 0.36 g dw L^−1^ OD_660_^−1^, which is typical for *P. denitrificans* in small batches. In this fed-batch, however, the cells and medium darkened as the experiment progressed, apparently skewing the OD_660_ reads. A direct dry weight measurement at 188 h indicated a conversion factor of 0.26 g dw L^−1^ OD_660_^−1^. Corrected OD-based estimates, assuming that this conversion factor is valid throughout the experiment are also shown (closed circles). Transient periods of exponential growth are indicated as well as the first manual injection of trace elements (TRES-2) at 71 h. The growth rates were estimated by nonlinear regression of biomass against time. The bottom show measured concentrations of NO_3_^−^ and NO_2_^−^

The observed cell yield, as determined by OD_660_ measurements, appeared significantly higher than anticipated based on the total amounts of NO_3_^−^ and glucose added to the reactor. The discrepancy was tentatively attributed to a progressing coloration of the culture influencing OD_660_ readings. To acquire a more direct biomass estimate, subsamples (n = 3) collected at the end of cycle 1 were employed for dry weight determination. The resulting conversion factor of 0.26 g dw L^−1^ OD_660_^−1^ was in closer agreement with the expected yield as estimated based on the cumulative provision of glucose and NO_3_^−^ (Fig. 8).

A closer inspection of the growth kinetics during the first cycle shows periods of exponential growth, albeit at low rates. During the first 45 h, the fed-batch culture had an exponential growth rate of 0.074 h^−1^, but subsequently reached a near stand-still which lasted 24 h (Fig. 8). During this lag, the concentrations of glucose, NO_2_^−^ and NO_3_^−^ were low, measured at < 1 mM, < 50 µM, and < 1 µM, respectively. Between 66 and 71 h into the fed-batch, several actions were taken in a stepwise manner. First, at 66 h, the N_2_-sparging was adjusted from 0.02 to 0.5 L min^−1^. This led to an increase in the rate of acid pumping (Figure S4 in Additional File D), and an increase in estimated growth rate (µ) from 0.024 (63–66 h) to 0.082 h^−1^ (66–69 h). Although increased N_2_ sparging enhanced growth, we observed a spike in N_2_O increasing from below detection at 65 h (< 2 ppm) to 145 ppm in the headspace, while NO showed only a slight increase from 1.7 ppm to 4.4 ppm. The transient increase in N_2_O could indicate Cu-limitation. At 69.5 h glucose was dosed to 1.25 mM in the reactor to ensure adequate C availability before trace elements (TRES-2) were provided at 71 h. The addition of glucose and trace elements did not further enhance growth (apparent µ_69-71h_ = 0.085; µ_71-73h_ = 0.083, estimated based on the rate of acid provision), but after 73 h, N_2_O had dropped below detection (Fig. 7).

The growth rate after the upshift in N_2_ sparging at 66 h, as estimated by the observed increase in biomass, reflected the enhanced rates of acid provision, transiently reaching 0.074 h^−1^ (Fig. 8). During the first 1–2 h of this growth phase, NO_3_^−^ was detectable in the medium at up to 0.34 mM, whereas NO_2_^−^ accumulated to a maximum of 3.1 mM. Both subsequently dropped below 1 µM. It appears likely that the 24 h lag phase starting at 45 h was chiefly due to insufficient N_2_-sparging, leading to CO_2_ + HCO_3_^−^ accumulation hence acidification of the medium, which in turn restricted the provision of C, N, and e^−^ -acceptor. Moreover, a limitation in Cu was likely the cause of lower activity by the Cu-dependent N_2_O reductase, leading to N_2_O accumulation.

Although we were able to transiently enhance growth in the reactor, the maximum growth rates never approached those observed in low cell density cultures. To query if the growth restrictions could be due to TE limitations/toxicity or accumulation of other toxic/inhibitory compounds, cell-free samples (supernatants after centrifugation to remove the cells) taken at various time points throughout the cultivation were frozen and later analyzes by HPLC, headspace-GC, and ICP-MS. The results suggested that growth could be Cu− and Fe− limited during the early lag phase in cycle 1 (~ 45–70 h), since the concentration of both elements was below detection at 70 h. However, the same did not hold for subsequent lag phases, where TEs appeared to be available in ample amounts (Additional File D). On the flip side is the potential for metal toxicity, particularly for Cu and Fe. Hahnke et al. [29] showed that *P. denitrificans* growing anaerobically was unaffected by 2.8 µM Cu^2+^, however, concentrations in the reactor reached 7.1 µM towards the end of cycle 1 and accumulated to even higher concentrations in cycle 2, which could potentially be inhibiting. Fe(II) is highly reactive and easily oxidized to Fe(III) with O_2_ or N-oxides as electron acceptors. The oxidation can be purely chemical or biological, but in both instances, they can lead to the formation of toxic radicals by Fenton reactions [46]. The initial iron in the reactor (15 µM) was almost entirely taken up by the cells within the first 70 h. The first injection of the trace element solution restored the concentration to 11 µM Fe (measured at 91 h), but the subsequent injections resulted in an overshoot causing a concentration of 110 µM Fe at the end of cycle 1 (189 h), while the maximum observed Fe concentration in cycle 2 exceeded 300 µM (Figure S5 in Additional File D). Based on these results, our initial measurement of iron content in anaerobically growing cells of 6.12 mg g^−1^ (0.6% of dry weight; Fig. 5) might have been the result of uncontrolled uptake from a Fe-rich medium rather than actual requirements. Tortell et al. [47] reported that heterotrophic bacteria under iron-sufficient conditions contained 0.1 mg Fe g^−1^. When inspecting the apparent uptake of TEs in Fed-batch 2 based on the cumulative amounts provided vs measured concentrations in liquid during the first 92 h, we found that the estimated Fe content in cells was similar to that found in cells from Fed-batch 1 and comparable to that reported by Tortell et al. [47] (Table 3). This, and the observed accumulation of Fe after the initial 70 h of Fed-batch 2 suggest that our trace element solution (TRES-2) exceeded the organism’s Fe requirements.

**Table 3:** Trace element concentrations in cells from a low-density batch Bioassay 1) and two high cell density cultivations, Fed-batch 1 and 2

Element	Content (µg g^−1^) in low cell-density batches during anaerobic growth (Bioassay 1) (n = 3)	Content (µg g^−1^) after high-cell density cultivation (Fed-batch 1) (n = 2)	Estimated content (µg g^−1^) in high-cell density cultivation (Fed-batch 2, 0–92 h) during exponential growth			
			OD = 12	OD = 36.2	OD = 43.2	OD = 44
Co	33 ± 1	3.8 ± 0.1	8.2	4.9	4.1	4.9
Cu	540 ± 70	16.5 ± 0.2	26.5	24.3	20.3	26.8
Fe	6000 ± 1000	195 ± 2	299	216	181	184
Mn	920 ± 70	62 ± 2	96.7	74.2	61.9	69.2
Mo	110 ± 20	6.3 ± 0.2	28.9	11.5	9.6	12.1
Ni	1.3 ± 0.2	12.9 ± 0.3	–	–	–	–
Zn	10,000 ± 1000	128.7 ± 0.3	560	170	140	144

The trace element contents in the cells from Bioassay 1 and Fed-batch 1 were measured directly in the biomass, whereas the TE contents in Fed-batch 2 cells were estimated based on cell density and apparent uptake (added amounts of each element subtracting that measured in the liquid).

HPLC and HS-GC did not reveal any adverse components in the reactor liquid. In defined medium, even at high cell density, acetaldehyde was the only metabolite detected (14.1 µM), whereas after the addition of yeast extract (1 g L^−1^) 260 h into the fed-batch, low µM concentrations of ethanol, acetone, diacetyl, 2- and butanol, and 0.76 mM α-ketoglutaric acid appeared (Table S3 in Additional File D).

Although the observed concentrations of metabolites were not likely to cause inhibition, the accumulation of trace metals could potentially be toxic. To ascertain whether inhibitory or toxic compounds in the medium caused the slow growth, or if the lag was due to physiological constraints within the cells, we harvested the reactor liquid at 330 h, separated cells and medium by centrifugation, and conducted a series of small-batch experiments (Bioassays 3–4).

#### Bioassays 3–4: no accumulation of toxic or inhibitory compounds, but reduced fitness of cells

**Fig. 9:**
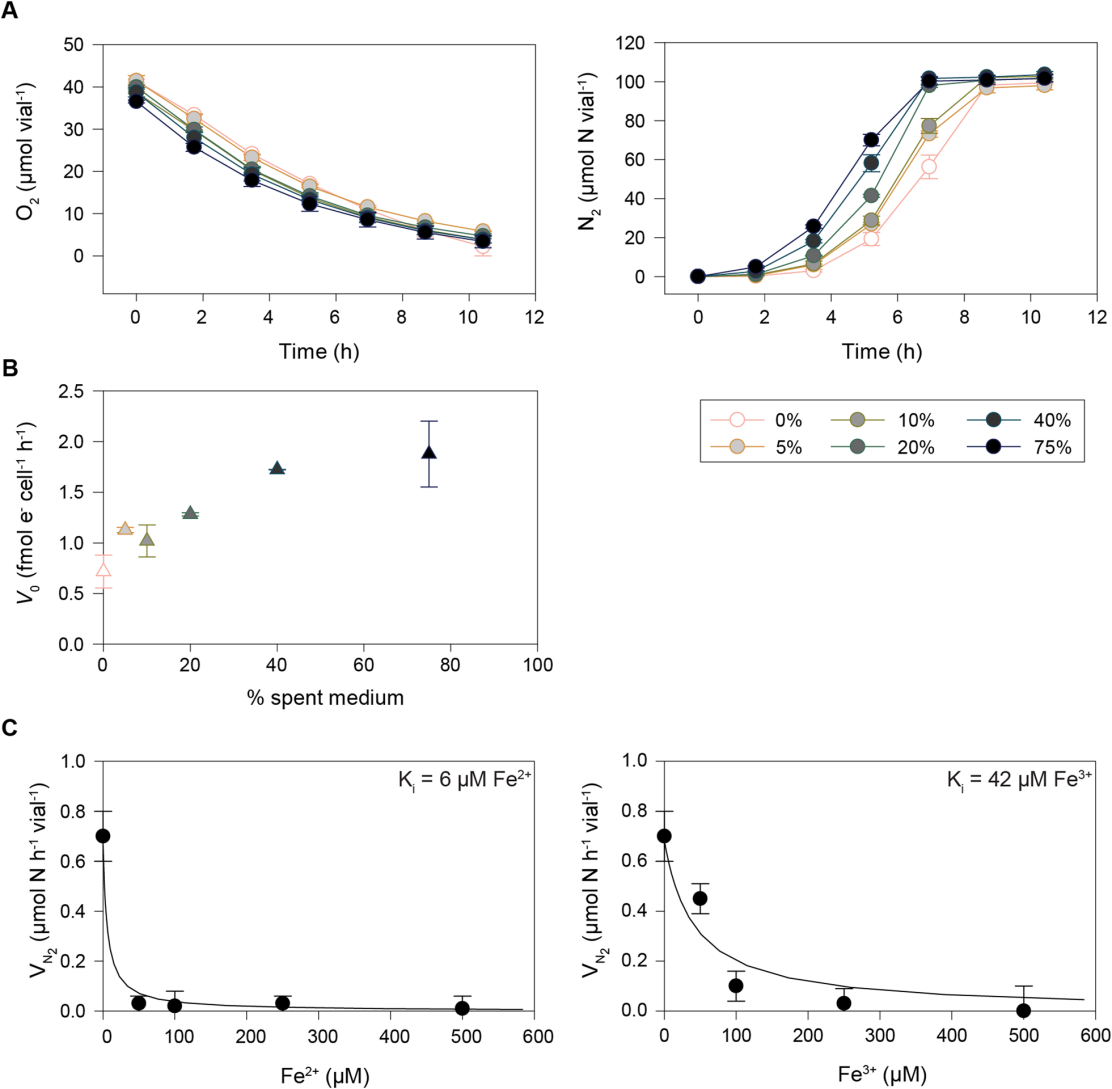
Assessment of supernatant toxicity in Bioassay 3. Fresh cells were inoculated in bioreactor liquid (0, 5, 10, 20, 40, and 75 vol%) in Bioassay 3a. **A** Gas data for each of the treatments (n = 2). The top shows the O_2_ concentration (µmol vial^−1^) while the bottom shows the cumulative N_2_ concentration (µmol N_2_-N vial^−1^). **B** Initial electron flow rate (fmol e^−^ cell^−1^ h^−1^) estimated for each of the treatments in Bioassay 3a, corrected for individual sampling time for each vial, assuming a linear increase in rate during the time (1.7 h) from the first to the second headspace sampling.** C** Bioassay 3b investigated the toxicity of Fe^2+^ and Fe^3+^. The average N_2_ production rate 10–34 h after injection of Fe^2+^/Fe^3+^ was calculated for each Fe concentration and plotted against the concentrations. Since [S] > > K_m_ both for glucose and nitrate, the inhibition coefficient (*K*_*I*_, i.e. the inhibitor concentration causing 50% inhibition) could be estimated by fitting *V* = *V*_*max*_ / (1 + [I]/*K*_*I*_) to the data by least square ([I] is the concentration of the inhibitors Fe^2+^ or Fe^3+^). The estimated *K*_*I*_ values are shown

Cell-free liquid and cells from the end of Fed-batch 2 were used to test for the accumulation of inhibitory compounds in the medium and the condition of the cells, respectively. To test the reactor liquid (Bioassay 3a), aerobically raised cells of *P. denitrificans* were transferred from serum vials to vials with increasing amounts of bioreactor liquid (0, 5, 10, 20, 40, and 75 vol%), 2 mM KNO_2_ and 1% O_2_ in duplicate serum vials containing 5 mL filter sterilized Sistrom’s medium (10 × concentrated) and MQ water to give a final volume of 50 mL. The vials were monitored for gas kinetics in the robotized incubation system at 28 °C until depletion of O_2_ and NO_2_^−^. There was no observable effect on aerobic respiration, and the reactor supernatant appeared to have a modestly stimulating effect on denitrification (Fig. 9). This, in combination with the results from HPLC and HS-GC (see Table S3 in Additional File D), led us to conclude that *P. denitrificans* itself does not excrete inhibitory compounds, even at high cell density. Moreover, with the exception of Fe(II), toxicity from the trace metals accumulated in the medium could be excluded as the reason for the low growth rates in the reactor.

Fe(II) is rapidly oxidized when exposed to oxygen. The separation of cells and reactor supernatant was done under ambient conditions, and Bioassay 3a was initially semi-aerobic. Thus, Fe would most likely exist as the less toxic Fe(III). To estimate and compare the K_i_ of Fe(II) vs Fe(III), we raised low-density anoxic cultures in basal medium and monitored their response (V_N2_, µmol N vial^−1^ h^−1^) to ~ 0, 50, 100, 250, and 500 µM Fe(II) (FeSO_4_) and Fe(III) (Fe(III)citrate) at pH ~ 7.5 (Bioassay 3b). As expected, the toxicity of Fe(II) was dramatically higher than Fe(III), with apparent K_i_ = 6 µM and 42 µM, respectively (Fig. 9C). Although crude, the test demonstrates the perils of iron provision and that care must be taken when designing TE solutions for HCDC.

**Fig. 10:**
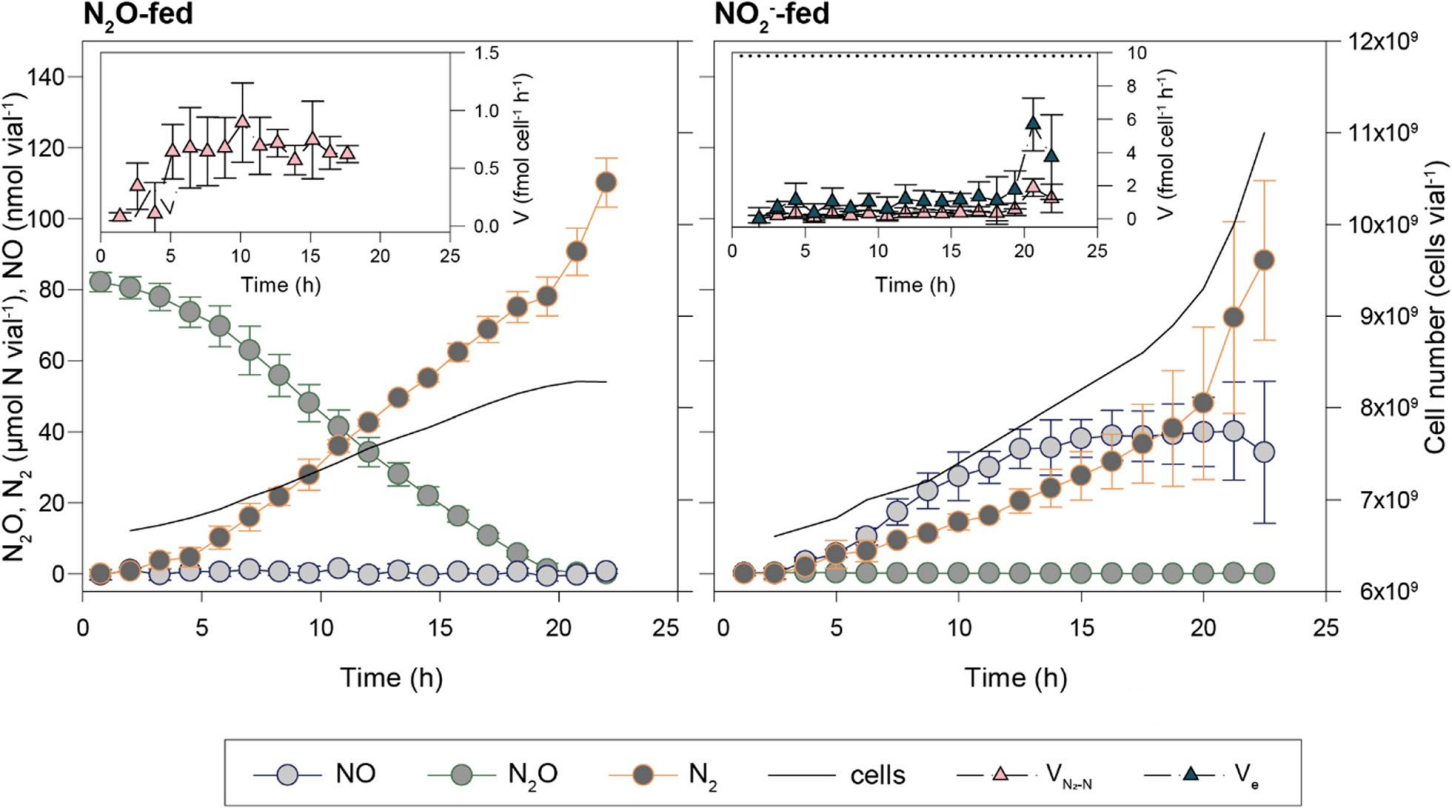
Reactor cells in fresh medium (Bioassay 4). Vials with He-atmosphere and 50 mL mineral medium with glucose were inoculated with cells (8·10^10^ cells vial^−1^) from the bioreactor and monitored for N gas kinetics while stirred at 30°C. Four vials were provided with N_2_O as the sole electron acceptor (~ 1 vol% N_2_O in the headspace = 41 µmol vial^−1^), while four vials were provided with NO_2_^−^ as the sole electron acceptor (2 mM KNO_2_ in the liquid). The large show the measured amount of NO, N_2_O, and N_2_ per vial (average, with standard deviation as vertical lines, n = 4), and the cell numbers per vial (calculated from the cumulated respiratory electron flow). The inserts show the cell-specific rates of N_2_-production (V_N2_, fmol N cell^−1^ h^−1^) and electron flow (V_e_, fmol e^−^ cell^−1^ h^−1^). For the N_2_O-fed cultures (left), only V_N2_ is shown because V_e_ equals V_N2_ (1 electron per N). The dotted line represents V_max_ in cells with µ = 0.19 h^−1^ assuming yield = 1.93 · 10^13^ cells mol^−1^ e^−^ to NO_3_- [27]

To test the physiological state of the cells in the Fed-batch 2, we inoculated HCDC cells in vials containing mineral medium (M1; 0.2 mL L^−1^ TRES-2) with 20 mM glucose and either 41 µmol N_2_O or 2 mM KNO_2_ (Bioassay 4). Within each treatment, two vials were inoculated with cells washed in PBS to ensure the complete removal of any inhibitory compound in the reactor liquid or attached to the cell surface, and two vials were inoculated with cells undergoing the same wash procedure, however, the pellet was resuspended in the same reactor liquid as a control. There was no significant difference between the cells washed in PBS and the control, so the results reported are based on the averages for all four cultures provided with N_2_O and NO_2_^−^, respectively. The gas kinetics showed that the cells were severely impaired in terms of respiration rates (Fig. 10). In the cultures provided with NO_2_^−^, the cell-specific electron flow rate to terminal acceptors was ~ 1 fmol e^−^ cell^−1^ h^−1^, which is nearly an order of magnitude lower than that expected for healthy cells under these conditions (V_max_ = 9.8 fmol e^−^ cell^−1^ h^−1^ assuming µ = 0.19 h^−1^ (Table 2) and yield = 1.93 · 10^13^ cells mol^−1^ e^−^ to NO_3_^−^; observed initial rate for M1 cultures in Bioassay 1: 6.9 ± 0.2 fmol e^−^ cell^−1^ h^−1^, assuming 1.25 · 10^9^ cells mL^−1^ OD_660_^−1^). After 20 h, the cultures appeared to recover, approaching rates comparable to healthy cells. The cells fed with N_2_O did not have a nitrogen source for assimilation, likely negating recovery to normal rates, and the electron flow remained at around 0.5–1 fmol e^−^ cell^−1^ h^−1^ throughout the incubation.

The reactor cells were clearly unhealthy and based on Bioassays 3a and 3b (Fig. 9), it appears likely that Fe(II) and chemical reactions during TE-dosing (e.g. transient formation of toxic radicals from Fe(II) oxidation) could have had a long-lasting negative impact (apparent recovery after 20 h in the NO_2_-fed cultures, Fig. 10). Another factor likely to directly impact overall fitness is insufficient access to nitrogen for assimilation. Since HNO_3_ served as both the electron acceptor and N-source, the observed lag in reactor cells may, at least in part, be attributable to the effects of prolonged nitrogen restriction.

#### Uneven distribution of nitrite reductase in the fed-batch population

A direct indicator of a population’s potential for anaerobic respiration and growth would be the denitrification proteome pool, of which nitrite reductase (Nir) is a key enzyme. The *P. denitrificans* strain used in Fed-batch 2 carries a *mCherry-nirS* fusion gene replacing the native *nirS* [28], and the expression of Nir in single cells could thus be gauged by fluorescence microscopy.

**Fig. 11:**
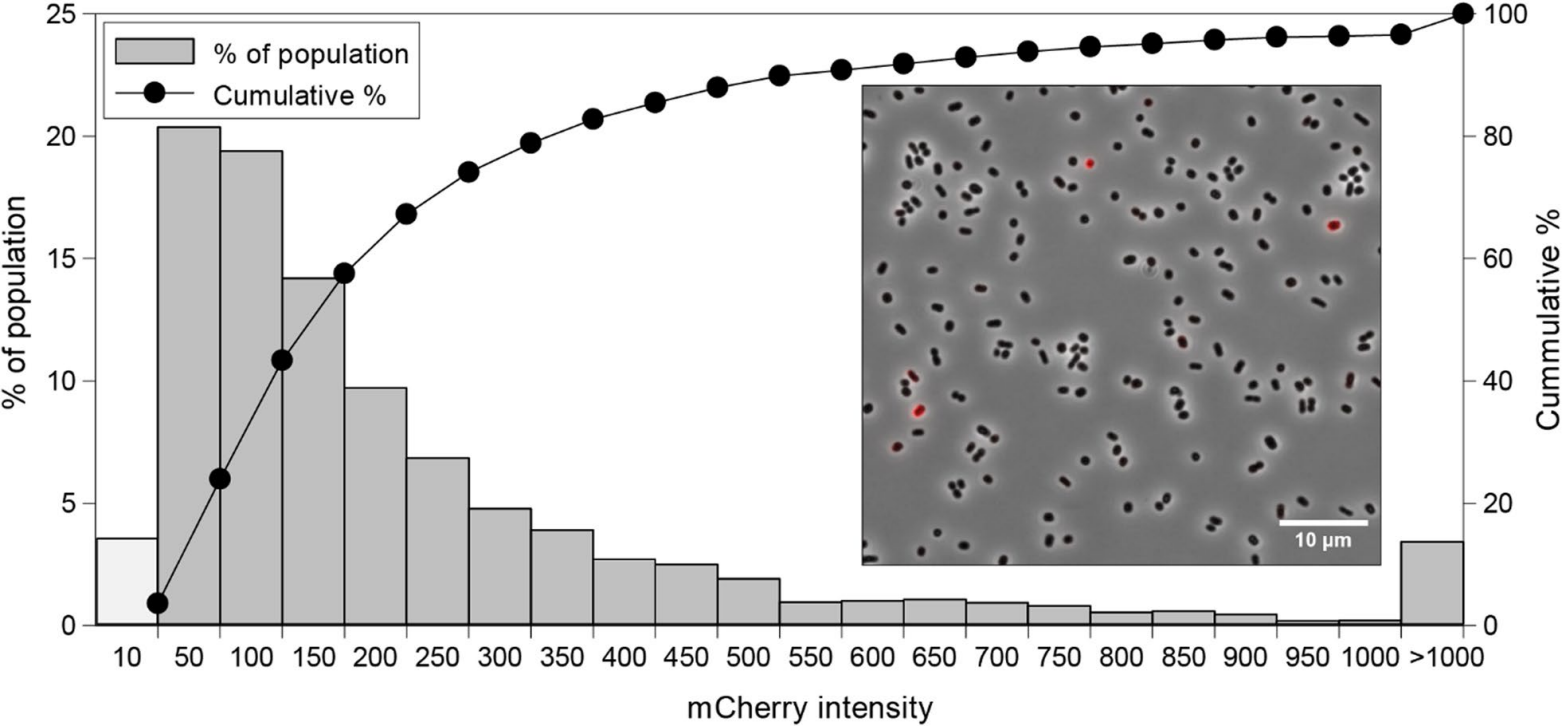
mCherry-NirS fluorescence in cells after 165 h. Images were obtained and analyzed as in Lycus et al. [28]. Fluorescence in each cell (n = 3769) was corrected for background and plotted as a histogram on the left y-axis (the number below shows the upper limit for each bar) and as the cumulative percentage in each bin on the right y-axis. The light grey is negative cells (≤ 10; average signal in negative controls: ~ 5). The insert shows one of the fluorescent images (phase contrast (grey) and mCherry (red) channels were merged in ImageJ), revealing very weak mCherry intensity in most of the cells

*P. denitrificans* (both the parent strain Pd1222 and the *mCherry-nirS* mutant) is known to express Nir only in a fraction of cells during transition from oxic to anoxic conditions, but Nir is expected to be expressed in all cells after several generations of anaerobic growth [28]. Contrary to this, fluorescence microscopy of cells sampled after 165 h showed high expression of Nir in only a minor subpopulation, the most conservative estimate being as low as 4% of the total population (fluorescence intensity > 1000; Fig. 11). The apparently low Nir pool in most cells would be expected to lead to NO_2_^−^ accumulation in a system with a comparably large and active pool of nitrate reductase (Nar). It may then seem surprising that NO_2_^−^ accumulated only to low mM concentrations in episodes (Figs. 7 and 8, e.g. 70–100 h, 210–250 h), suggesting that V_NIR_ matched V_NAR_ most of the time. The limited NO_2_^−^ accumulation did not necessarily reflect equal pools of Nir and Nar, however, but was more likely due to insufficient provision of HNO_3_, which allowed Nir to keep pace with Nar in the culture as a whole. Whether the heterogeneity with respect to Nir expression would also develop in a fed-batch culture with ample provision of NO_3_^−^ is an open question, but previous data showing even Nir expression in well-adapted small batches [28], suggest that this would not occur to any large extent. Nevertheless, when selecting organisms for anaerobic HCDC, strong expression of all the denitrification enzymes would be a qualifier to take into consideration.

### Fed-batch 3: Sustained high cell density cultivation in repeated cycles

**Fig. 12:**
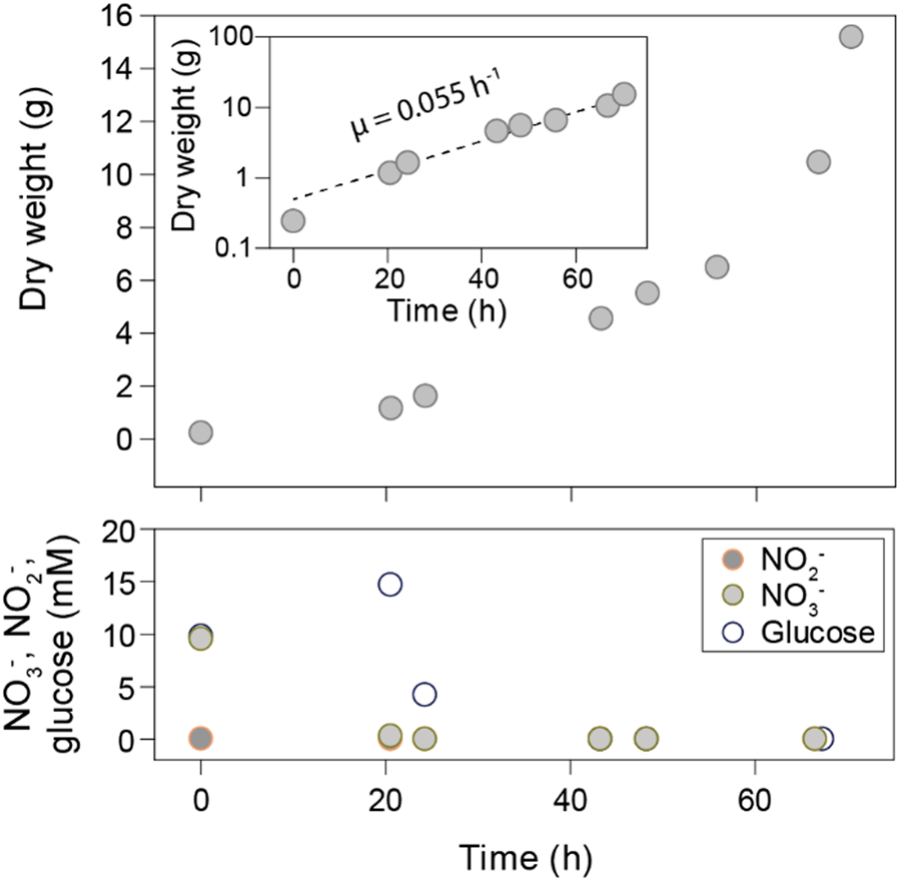
Growth rate and liquid analysis during the first cycle of Fed-batch 3. The top shows the total dry weight in the reactor, which was found by considering the liquid volume in the reactor during each OD_660_ measurement and assuming 0.36 g dw OD_660_^−1^ L^−1^. The insert shows the apparent specific growth rate throughout the cycle. The lower shows the offline measurements of NO_2_^−^, NO_3_^−^, and glucose

Two of the take-home messages from Fed-batch 2 were that: (1) exponential growth was not easily achieved at OD_660_ > 65–70; and (2) we had overestimated the organism’s requirement for Fe. Thus, Fed-batch 3 was run in four consecutive cycles (240 h total), each up to an OD_660_ of 46 and with a trace element composition where Fe had been reduced to 1/5 of the concentration in TRES-2 used in Fed-batch 2 (TRES-3, Table 1). Each of the four cycles was initiated by replacing most of the reactor liquid with fresh basal medium (Figure S6 in Additional File E). In the first cycle, the growth rate was 0.055 h^−1^, and at the time of harvest, the total dry weight in the reactor was 15.2 g (16.68 g L^−1^) (Fig. 12). The remaining three cycles had growth rates of 0.030, 0.037, and 0.040 h^−1^. All four cycles showed somewhat lower growth rates than the maxima of Fed-batch 2 (Fig. 8) but still demonstrated the potential for maintaining exponential growth to high density through repeated batch cycles.

**Fig. 13:**
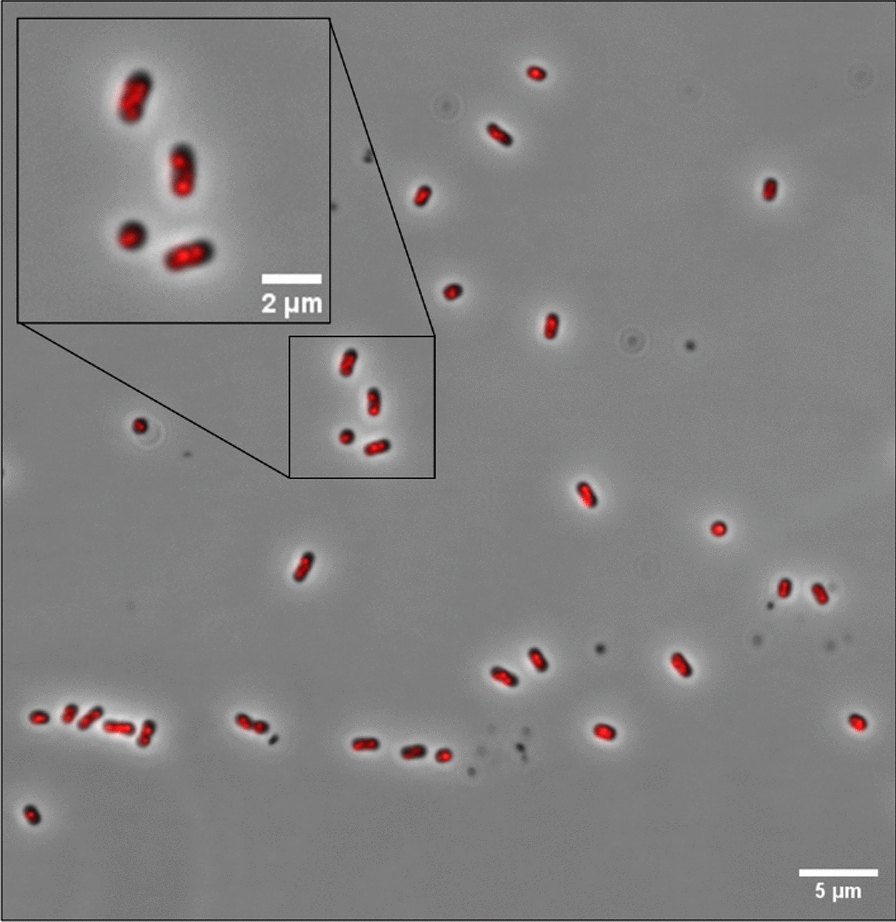
Reactor cells stained with Nile red. The cells were harvested at the end of the reactor run, stained with Nile red, and visualized by fluorescence microscopy (phase contrast (grey) and Nile red (red) channels were merged in ImageJ). The presence of PHA granules was visible in all cells

At the end of the last cycle, the content of the bioreactor was collected. Subsamples were retrieved for microscopy before the rest of the cells were harvested by centrifugation and freeze-dried. Kjeldahl-N was used for determination of total organic N and NH_4_^+^/NH_3_. The protein content was found to be 42% (66.43 g kg^−1^ Kjeldahl-N), which is lower than expected for bacterial cells. A plausible explanation for this could be accumulation of PHA. To assess whether this was the case, a sample was stained with Nile red for the detection of PHA granules. Inspection by fluorescence microscopy indicated PHA accumulation throughout the population (Fig. 13).

The concentration of nitrogen oxyanions was low throughout the process, which may have triggered storage of available C as PHA. Despite being a valuable resource for bioplastics production [48], high PHA concentrations are undesirable when the primary aim is to maximize protein content. In our reactor runs, the near absence of NO_3_^−^ and excess of glucose has most likely caused the PHA accumulation, and we expect that an optimized and balanced provision of C and electron acceptor, or introducing periods of C-limitation, will resolve the issue.

### Final considerations

Microbial protein is emerging as a novel source of food and feed, but existing methods for HCDC depend on oxygen, which becomes a limiting factor in most processes due to its low water solubility. Here we have qualified oxygen-independent HCDC by denitrification. We have shown that the model organism *P. denitrificans* can reach densities > 20 g L^−1^ in a pH–stat in a defined medium with glucose as C-source and HNO_3_ as N-source and electron acceptor. The biomass yield was in fair agreement with the predicted yield from the cumulative consumption of glucose and HNO_3_, no adverse metabolic products accumulated in the liquid, even at high cell densities, and the trace metal provision was progressively adjusted to suit the organism. Initial fed-batch experiments informed a final run where *P. denitrificans* was repeatedly raised to > 15 g L^−1^ in four batch cycles, at reasonable growth rates.

We have pinpointed challenges to be addressed for moving forward. One is the accumulation of storage polymers such as PHA, leading to decreased protein yield, which we predict will be resolved in a process with ample electron acceptor availability and a carefully balanced provision of C. The most profound hurdle at this stage is the unexpectedly low growth rates, which never exceeded 50% of that observed in small-batch. Since we have ruled out trace element limitation and accumulation of inhibitors or toxic agents, we tentatively conclude that the main culprit is slow removal of CO_2_ from the medium. In water, CO_2_ forms carbonic acid (H_2_CO_3_), lowering the pH, which in turn shifts the equilibrium towards gaseous CO_2_. In our system, the formation of H_2_CO_3_ would counteract the denitrification-driven alkalization which triggers the provision of HNO_3_. The culture is thus likely to oscillate between spurts of respiration and depletion of NO_3_^−^ followed by growth arrest due to N and e^−^-acceptor deprivation. A critical aim in the further optimization of anaerobic HCDC will be to maintain a continuous availability of NO_3_^−^ in mM concentrations. This can be approached via several routes: (1) by selecting acidophilic denitrifiers facilitating operations at below-neutral pH, shifting the equilibrium towards gaseous CO_2_, thus enhancing the release from the liquid; (2) decreasing the solubility of gases by increasing the temperature and running the process at sub-atmospheric pressure; (3) applying a dynamic, CO_2_-responsive pH setpoint, ensuring that NO_3_^−^ is maintained at mM concentrations in the culture.

Although still in its early stages, anaerobic HCDC has been proven viable, and may become a feasible option for single-cell protein production for feed and food. Moreover, it may be used for efficient production of compounds that are sensitive to O_2_ and reactive oxygen species (ROS).

## Supplementary Information

Additional file 1. A. Determining growth rates on glucose with forced nitrate assimilation. The file contains details about the experiment for determination of growth rates, biomass yields, and trace element uptake. Figure S1 shows the gas kinetics for aerobic growth in Sistrom’s medium and the M1 and M2 media. Table S1 shows the full dataset for Figure 5, which is the trace element composition in the medium and the cells following aerobic and anaerobic incubations

Additional file 2. B. Fed-batch 2 The file contains a description of Fed-batch 1. Figure S2 shows a summary of Fed-batch 1 with a description of the different phases in the experiment

Additional file 3. C. Batch experiments for testing pump reservoir solutions. The file details the experimental setup for the batch experiment for testing reservoir solutions. Table S2 shows the experimental setup. Figure S3 shows the result from the test of the acid + ME mixture and the test of the TRES-2 solution

Additional file 4. D. Fed-batch 2. The file details the experimental setup of Fed-batch 2. Figure S4 shows the response of changing the N_2_ sparging flow rate and glucose and TRES-2 injections between 63–73 h. Table S2 shows the result of HPLC and headspace-GC analysis of volatile fatty acids and other metabolites in supernatant samples taken throughout the fed-batch. Figure S5 shows the ICP-MS result of the supernatant samples.

Additional file 5. E. Fed-batch 3. The file details the experimental setup of Fed-batch 3. Figure S6 shows the overview of the fed-batch including offline measurements

## Data Availability

Crucial data are included in this published article and its additional files and other data can be made available from the corresponding author upon reasonable request.
