# Supplementary material for: High cell density cultivation by anaerobic respiration
Source: Microb Cell Fact. 2024 Nov 25;23:320. doi: 10.1186/s12934-024-02595-8 (PMC11590539; doi:10.1186/s12934-024-02595-8)
Supplement: Supplementary file 1 — Additional file 1. A. Determining growth rates on glucose with forced nitrate assimilation. The file contains details about the experiment for determination of growth rates, biomass yields, and trace element uptake. Figure S1 shows the gas kinetics for aerobic growth in Sistrom’s medium and the M1 and M2 media. Table S1 shows the full dataset for Figure 5, which is the trace element composition in the medium and the cells following aerobic and anaerobic incubations [file 12934_2024_2595_MOESM1_ESM.docx]

Additional File A

High Cell Density Cultivation by Anaerobic Respiration

Marte Mølsæter Maråk^1^, Ricarda Kellermann, Linda Liberg Bergaust^1*^ and Lars Reier Bakken^1^.

*^1^Norwegian University for Life Sciences, Faculty of Biotechnology, Chemistry and Food Science*

*^*^Corresponding author:* [linda.bergaust@nmbu.no](mailto:linda.bergaust@nmbu.no)

Determining growth rates on glucose with forced nitrate assimilation

Bioassay 1 was designed to determine the maximum growth rates and growth yields for aerobic and anaerobic growth on glucose, depending on the form of nitrogen assimilated (NH_4_^+^ or NO_3_^-^). This was done using a mineral base medium with trace element composition TE-1 (Table 1 in the main text) supplemented with glucose (1.45, 2.9, or 5.8 mM) and 10 mM NO_3_^-^, with and without 3.6 mM NH_4_^+^ (M2 and M1**,** respectively). In some vials, a second dose of 10 mM NO_3_^-^ was injected after the first 10 mM was depleted. We included vials with Sistrom’s medium containing 34 mM succinate and 3.6 mM NH_4_^+^ as a control.

*P. denitrificans* was first raised from frozen stocks by aerobic batch cultivation in each medium overnight. The cultures were then used as inoculum (giving an initial OD_660_=0.001) to vials with 7 vol% O_2_ in the headspace (Figure S1). The electron flow to oxygen was used to estimate the aerobic growth rate in each medium. Once the cultures had depleted both the O_2_ and NO_3_^-^, the cultures were used to inoculate new anaerobic vials (initial OD_660_=0.001) with the same media and different glucose concentrations (n=3 for each combination). The residual trace element composition in the medium and the content in the cells following aerobic and anaerobic growth in the M1 medium were found by ICP-MS (Table S1).


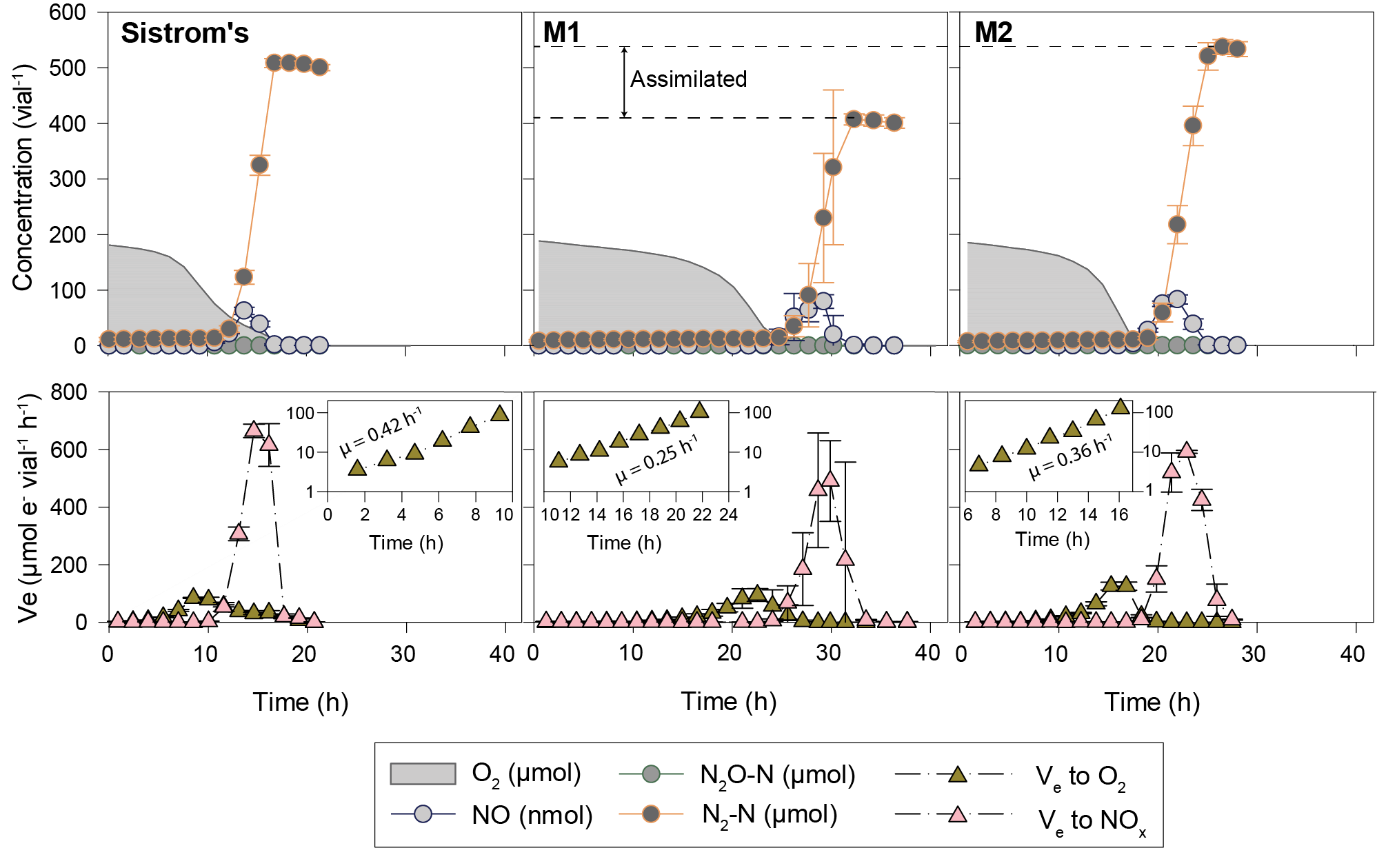


**Figure S1 Gas kinetics in batch cultures during the transition from aerobic respiration to denitrification*.*** *P. denitrificans* was inoculated in vials containing Sistrom’s, M1, or M2 medium supplemented with 7% O_2_, 10 mM NO_3_^-^ and 11.6 mM glucose. The cells growing in M1 (no NH_4_^+^) assimilated approximately 15% of the available NO_3_^-^. Aerobic growth rates were estimated by regression of the electron flow to oxygen during exponential aerobic growth as seen in the insert.

**Table S1 Trace element content in cells and liquid following anaerobic and aerobic batch incubations.**

|  | **Liquid concentration (µM), n=3** | | | **Cell content (mg g^-1^), n=3** | |
| --- | --- | --- | --- | --- | --- |
|  | **Initial** | **After aerobic incubation** | **After anaerobic incubation** | **After aerobic incubation** | **After anaerobic incubation** |
| **Al** | 1.1 ± 0.6 | 0.8 ± 0.1 | 1.95 ± 0.09 | 0.43 ± 0.06 | 1.0 ± 0.1 |
| **Ba** | 0.011 ± 0.001 | n.d. | 0.0085 ± 0.0004 | 0.025 ± 0.003 | 0.044 ± 0.006 |
| **Co** | 2.25 ± 0.04 | 1.9 ± 0.1 | 2.05 ± 0.02 | 0.044 ± 0.008 | 0.033 ± 0.001 |
| **Cu** | 1.40 ± 0.03 | 1.14 ± 0.08 | 0.13 ± 0.01 | 0.13 ± 0.02 | 0.54 ± 0.07 |
| **Fe** | 5.3 ± 0.5 | 0.3 ± 0.1 | 0.9 ± 0.5 | 9 ± 3 | 6 ± 1 |
| **Mn** | 8.1 ± 0.1 | 5.1 ± 0.2 | 5.8 ± 0.2 | 1.0 ± 0.2 | 0.92 ± 0.07 |
| **Mo** | 0.82 ± 0.01 | 0.75 ± 0.01 | 0.78 ± 0.03 | 0.14 ± 0.02 | 0.11 ± 0.02 |
| **Ni** | 0.080 ± 0.003 | 0.07 ± 0.01 | 0.078 ± 0.003 | 0.0030 ± 0.0004 | 0.0013 ± 0.0002 |
| **Sr** | 0.29 ± 0.01 | 0.22 ± 0.00 | 0.22 ± 0.03 | 0.042 ± 0.008 | 0.066 ± 0.008 |
| **Zn** | 20.9 ± 0.9 | 7.7 ± 0.4 | 12 ± 1 | 11 ± 2 | 10 ± 1 |

*The table shows the measured TE concentration in the medium before and after aerobic and anaerobic incubations (µM, n=3), and the content in cells (mg g^-1^, n=3) after the incubations.*
