# Supplementary material for: High cell density cultivation by anaerobic respiration
Source: Microb Cell Fact. 2024 Nov 25;23:320. doi: 10.1186/s12934-024-02595-8 (PMC11590539; doi:10.1186/s12934-024-02595-8)
Supplement: Supplementary file 2 — Additional file 2. B. Fed-batch 2 The file contains a description of Fed-batch 1. Figure S2 shows a summary of Fed-batch 1 with a description of the different phases in the experiment [file 12934_2024_2595_MOESM2_ESM.docx]

Additional File B

High Cell Density Cultivation by Anaerobic Respiration

Marte Mølsæter Maråk^1^, Ricarda Kellermann, Linda Liberg Bergaust^1*^ and Lars Reier Bakken^1^.

*^1^Norwegian University for Life Sciences, Faculty of Biotechnology, Chemistry and Food Science*

*^*^Corresponding author:* [linda.bergaust@nmbu.no](mailto:linda.bergaust@nmbu.no)

Fed-batch 1

The bioreactor was filled with 1 L of mineral base medium with TE-1, supplemented with 10 mM NO_3_^-^ and 55.6 mM glucose. The acid and the macro-(ME) and trace-(TE) elements were mixed in one reservoir (5 M HNO_3_, 0.875 g L^-1^ MgSO_4_ · 7H_2_O, 0.175 g L^-1^ CaCl_2_ · 2H_2_O, 3.45 g L^-1^ KH_2_PO_4_, and 26.1 g L^-1^ K_2_HPO_4_, TE-1), and the pumping of this was controlled by pH, while glucose was added manually twice before being mixed with the acid reservoir after 192 hours. We also added extra NO_3_^-^ manually twice during the initial phase, in response to apparent NO_3_ limitations. Sparging of the reactor was initiated after 172 h to lower the partial pressure of CO_2_.

Due to various manipulations and changes throughout the batch, the experiment can be divided into 5 phases, all commented and discussed in the legend (Figure S2). During the initial phase (A), growth was exponential, but paused after 38 hours, possibly due to NO_3_^-^ limitation since growth was restored after manual acid injection. There was a period (B) of unbalanced denitrification, where high concentrations of NO_2_^-^ and N_2_O were measured. Then followed a period of growth arrest (C), presumably because the high partial pressure of CO_2_ kept the pH below the pH setpoint for the acid pump, thereby causing NO_3_^-^ limitation. There was a transient period of growth after glucose was added to the acid and the pH setpoint was lowered slightly to trigger an injection. Growth resumed after the sparging was initiated (D), giving clear indications that high levels of CO_2_ had restricted the substrate provision. In the final stage (E), there was a growth arrest most likely caused by a gradual accumulation of toxic compounds by chemical reactions in the acid reservoir after the second addition of glucose (visual color change in the reservoir).


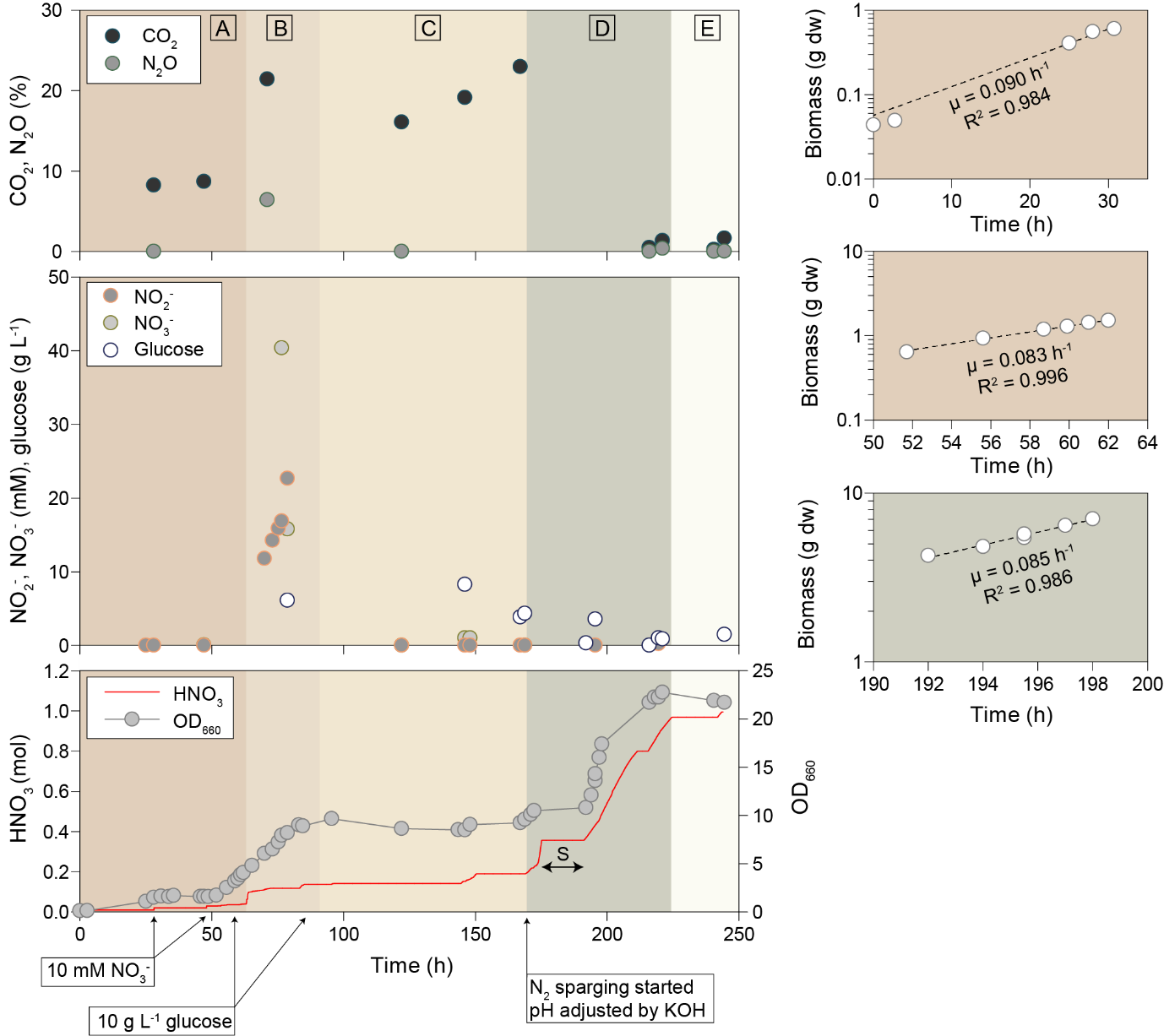


**Figure S2 Summary of the first attempt to grow cells to high densities in a pH-stat bioreactor (Fed-batch 1)**. Left panels: The top panel shows measured concentrations of gases in the headspace, the mid panel shows measured concentrations of NO_2_^-^, NO_3_^-^, and glucose, and the bottom panel shows cumulated input of HNO_3_ and measured OD_660_. The different phases of the experiment are indicated by color and letters (A-E). **A)** During the initial phase the cells were transitioning to denitrification, and as the density of cells and the rate of denitrification was too low to increase the pH above the setpoint, NO_3_ was injected manually. Growth was initially exponential (µ = 0.09 h^-1^), but paused after 38 h, possibly due to depletion of NO_3_^-^ as growth was restored after the second injection of 10 mM NO_3_^-^. **B)** Exponential growth continued, and the acid pump was triggered. Growth declined after 80 h, plausibly due to two phenomena: 1) unbalanced denitrification causing rampant accumulation of NO_2_^-^ (and N_2_O) and 2) NO_3_^-^-limitation because high CO_2_ concentrations suppress pH, and hence no pumping of HNO_3_. **C)** Practically no growth, plausibly due to high *p*_CO2_. Glucose was added to the acid reservoir and the pH setpoint was lowered after 149 hours which led to a period of transient growth. **D)** Sparging with N_2_ was initiated and the pH was manipulated by an injection of KOH, resulting in substantial growth, plausibly by removing CO_2_. The paucity in growth between 175 and 190 hours (S) was caused by an uncoupling of the acid pump. **E)** Mixing glucose into the reservoir with HNO_3_ and mineral elements (192 h) apparently generated toxic compounds which accumulated to toxic levels after 230 h: the color of the reservoir liquid had changed to deep green and growth in the bioreactor came to a halt. The right panels show the estimated growth rate during different periods. The total biomass was calculated based on OD_660_ measurements, the total volume in the bioreactor, and assuming 0.36 g dw L^-1^ OD_660_^-1^.
