# Supplementary material for: High cell density cultivation by anaerobic respiration
Source: Microb Cell Fact. 2024 Nov 25;23:320. doi: 10.1186/s12934-024-02595-8 (PMC11590539; doi:10.1186/s12934-024-02595-8)
Supplement: Supplementary file 3 — Additional file 3. C. Batch experiments for testing pump reservoir solutions. The file details the experimental setup for the batch experiment for testing reservoir solutions. Table S2 shows the experimental setup. Figure S3 shows the result from the test of the acid + ME mixture and the test of the TRES-2 solution [file 12934_2024_2595_MOESM3_ESM.docx]

Additional File C

High Cell Density Cultivation by Anaerobic Respiration

Marte Mølsæter Maråk^1^, Ricarda Kellermann, Linda Liberg Bergaust^1*^ and Lars Reier Bakken^1^.

*^1^Norwegian University for Life Sciences, Faculty of Biotechnology, Chemistry and Food Science*

*^*^Corresponding author:* [linda.bergaust@nmbu.no](mailto:linda.bergaust@nmbu.no)

Bioassay 2 for testing pump reservoir solutions

The visible chemical reaction observed when all reservoir components (HNO_3_, glucose, and trace element solution) were mixed in one reservoir at the end of the first fed-batch attempt (Additional File B) prompted us to investigate if toxic compounds were produced when mixing the different feed components (glucose, HNO_3_, macro- (ME) and trace elements (TE)).

We tested this in low cell density 50 mL batch cultures using the incubation robot. Eight parallel cultures of *P. denitrificans mCherry-NirS* were prepared in serum vials containing 50 mL modified mineral medium (M1 with TE-1) with 1% initial O_2_, 5 mM NO_3_^-^ and 10 mM glucose. After the depletion of O_2_ and transition to denitrification, various mixtures were injected (Table S2).

The first part of the experiment aimed to test a mixed feed reservoir (3.125 M glucose + TRES-2, solution left at room temperature for >1 month) and compare the growth rate against vials receiving equal concentrations of pure glucose solution. The result (Figure 6 in the main text) showed an immediate inhibition of growth in the cultures receiving the mixed feed solution. In the second part, the acid containing ME (5 M HNO_3_, 1.54 g L^-1^ MgSO_4_ · 7H_2_O, 0.77 g L^-1^ CaCl_2_ · 2H_2_O, 1.54 g L^-1^ K_2_HPO_4,_ and 1.54 g L^-1^ NaH_2_PO_4_) was compared with HNO_3_ without MEs (Figure S3). We also tested whether the trace element solution alone was toxic. Due to space limitations, this was done in the same cultures as the acid test after depletion of the HNO_3_. Then, the cultures received 5 mM KNO_3_ either as KNO_3_ or KNO_3_ + an equal dose of TRES-2 as during testing of the glucose mixture.

**Table S2 Overview of the different treatments for testing toxicity of various reservoir components.**

|  |  | **Injection 1** | **Injection 2** |
| --- | --- | --- | --- |
| **Testing if a mix of glucose and TRES-2 is toxic.** | **Control** | 2 mM KNO_3_  10 mM glucose |  |
|  | **Glucose + TE reservoir** | 2 mM KNO_3_  Glucose + TRES-2 |  |
| **Testing if a mix of HNO_3_ and ME is toxic (injection 1) and if TRES-2 alone is toxic (injection 2).** | **Control / TE** | 2 mM HNO_3_  10 mM glucose | 2 mM KNO_3_ + TRES-2 |
|  | **HNO_3_ + ME / Control** | 2 mM HNO_3_ + ME  10 mM glucose | 2 mM KNO_3_ |


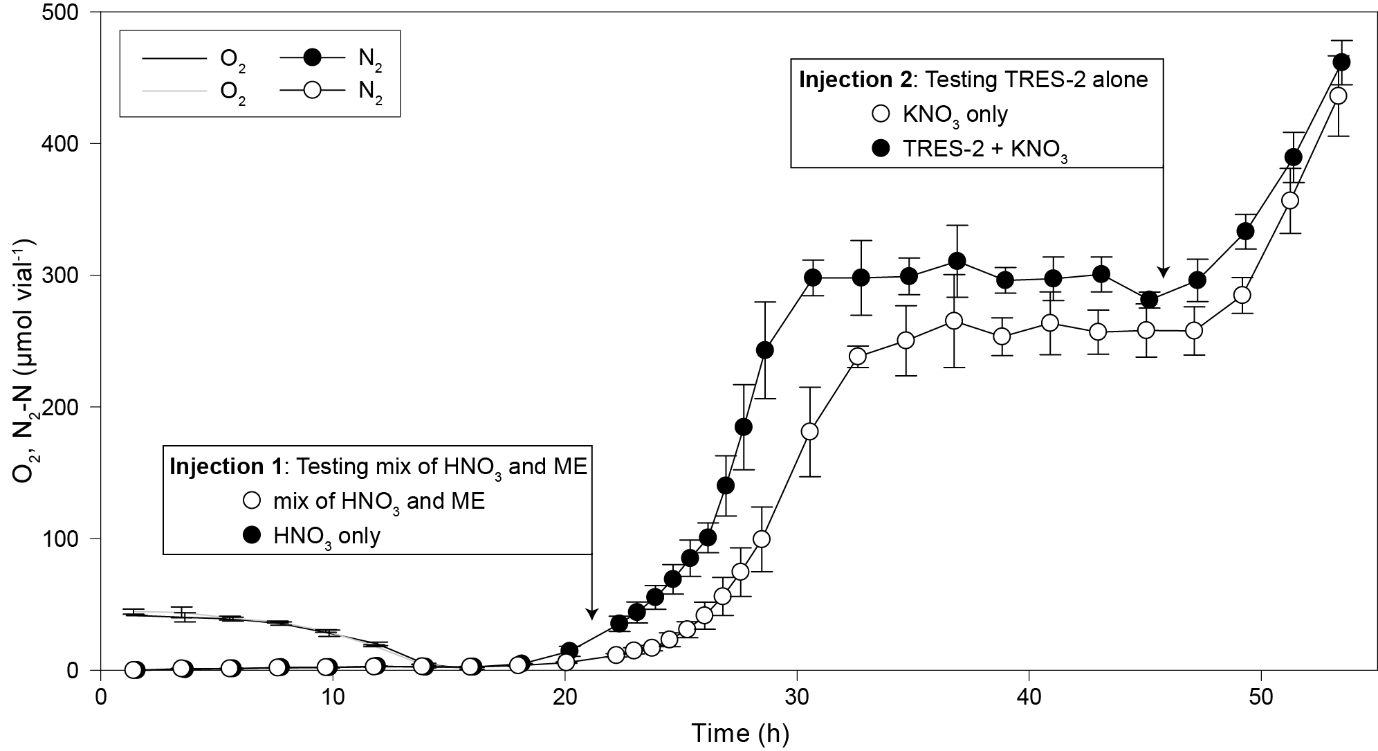


**Figure S3 Testing toxicity of the acid reservoir and the TRES-2 solution.** *P. denitrificans* was inoculated in serum vials (medium M1; initial 1 vol% O_2_ in the headspace, 5 mM KNO_3_, and 10 mM glucose). After depletion of O_2_ and onset of denitrification, all vials got an additional 10 mM glucose and 5 mM NO_3_^-^ as either HNO_3_ or HNO_3_ + ME (injection 1). After depletion of the NO_3_^-^, the vials received a second injection with either 5 mM KNO_3_ or KNO_3_ + TRES-2 (injection 2). The figure shows the O_2_ depletion (µmol O_2_ vial^-1^) and N_2_ production (µmol N_2_-N vial^-1^).
