# Supplementary material for: High cell density cultivation by anaerobic respiration
Source: Microb Cell Fact. 2024 Nov 25;23:320. doi: 10.1186/s12934-024-02595-8 (PMC11590539; doi:10.1186/s12934-024-02595-8)
Supplement: Supplementary file 4 — Additional file 4. D. Fed-batch 2. The file details the experimental setup of Fed-batch 2. Figure S4 shows the response of changing the N2 sparging flow rate and glucose and TRES-2 injections between 63–73 h. Table S2 shows the result of HPLC and headspace-GC analysis of volatile fatty acids and other metabolites in supernatant samples taken throughout the fed-batch. Figure S5 shows the ICP-MS result of the supernatant samples. [file 12934_2024_2595_MOESM4_ESM.docx]

Additional File D

High Cell Density Cultivation by Anaerobic Respiration

Marte Mølsæter Maråk^1^, Ricarda Kellermann, Linda Liberg Bergaust^1*^ and Lars Reier Bakken^1^.

*^1^Norwegian University for Life Sciences, Faculty of Biotechnology, Chemistry and Food Science*

*^*^Corresponding author:* [linda.bergaust@nmbu.no](mailto:linda.bergaust@nmbu.no)

Fed-batch 2

The bioreactor was filled with 500 mL of modified mineral medium (M1 with TE-2) with 10 mM glucose. The acid pump (reservoir solution: 5 M HNO_3_, 3.15 g L^-1^ MgSO_4_ · 7H_2_O, 0.77 g L^-1^ CaCl_2_ · 2H_2_O, 1.21 g L^-1^ K_2_HPO_4,_ and 1.55 g L^-1^ NaH_2_PO_4_) was triggered by lowering the pH setpoint to 7.4. The feed (3.125 M glucose) was triggered by a customized script so that for each mL of acid, 0.543 mL of feed was added, however, this value (k_feed_) was occasionally changed during the run (Figure 7 in the main text). The fed-batch was initiated by inoculation of 45 mL anaerobic culture of *P. denitrificans mCherry-NirS* (approximately 1 · 10^11^ cells). The stirring was initially set to 250 rpm but was adjusted several times during the experiment. Additional trace elements, glucose, and NO_3_^-^ were added manually. OD_660_, NO_3_^-^, NO_2_^-^, and glucose were analyzed from cell-free liquid samples, while CO_2_, N_2_O, and NO were analyzed in samples taken from the reactor headspace.

After 66 hours, several steps were taken to enhance the growth rate: 1) Increasing the sparging flow rate, 2) injecting glucose, and 3) injecting TRES-2 (Figure S4). Based on the increased frequency of acid injections it appears that the growth rate was increased following the increase in sparging flow rate.


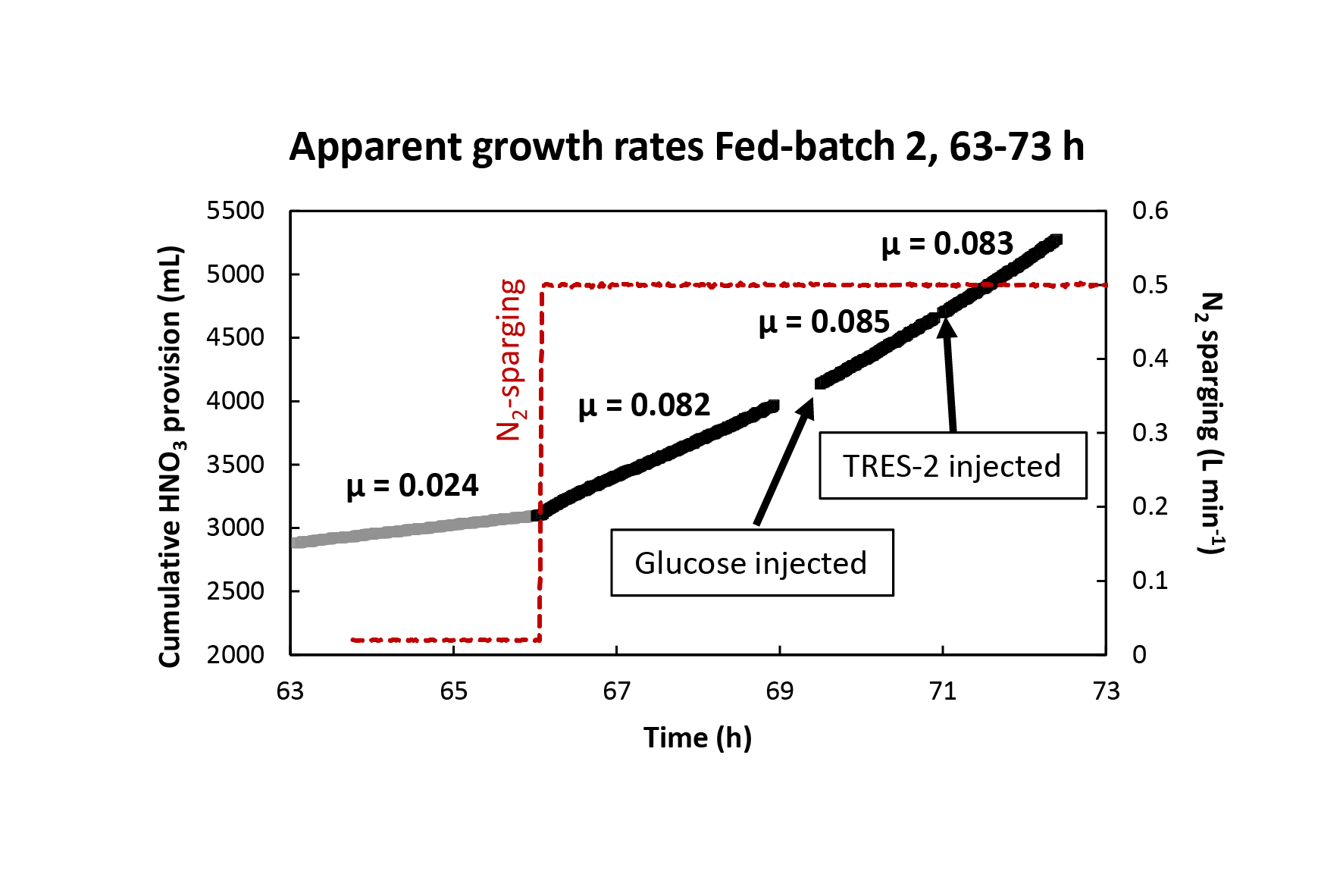


**Figure S4 Modification during Fed-batch 2 to increase growth rate.** After 66 hours the growth rate was increased to 0.5 L min^-1^, which resulted in an apparent increase in growth rate based on nonlinear regression of the cumulative HNO_3_ provision. The glucose injection and TRES-2 injection did not increase the growth rate substantially but did reduce the amount of N_2_O accumulation.

Cell-free liquid samples taken at irregular intervals during the reactor run were analyzed by headspace-GC and HPLC to quantify volatile fatty acids and metabolites (Table S3), and by ICP-MS to quantify trace element concentrations (Figure S5).

| **Time [h]** | **α-ketoglutaric acid [µM]** | **Glucose [µM]** | **Pyruvic acid [µM]** | **Lactic acid [µM]** | **Formic acid [µM]** | **Acetic acid [µM]** | **Acetaldehyde [µM]** | **Ethanol [µM]** | **Acetone [µM]** | **Diacetyl [µM]** | **2-butanol [µM]** | **Acetoin [µM]** |
| --- | --- | --- | --- | --- | --- | --- | --- | --- | --- | --- | --- | --- |
| **0** | n.d. | n.d. | n.d. | n.d. | n.d. | n.d. | 1.0 | 6 | n.d. | n.d. | 1.8 | n.d. |
| **17** | n.d. | n.d. | n.d. | n.d. | n.d. | n.d. |  |  |  |  |  |  |
| **65** | n.d. | n.d. | n.d. | n.d. | n.d. | n.d. |  |  |  |  |  |  |
| **119** | n.d. | 21648 | n.d. | n.d. | n.d. | n.d. |  |  |  |  |  |  |
| **161** | n.d. | 39792 | n.d. | n.d. | n.d. | n.d. |  |  |  |  |  |  |
| **188.9** | n.d. | 53949 | n.d. | n.d. | n.d. | n.d. | 14.1 | n.d. | n.d. | n.d. | n.d. | n.d. |
| **214** | n.d. | n.d. | n.d. | n.d. | n.d. | n.d. | 14.5 | n.d. | n.d. | n.d. | n.d. | n.d. |
| **330** | 763 | 72301 |  | n.d. | n.d. |  | 2.4 | 22 | 11 | 20 | 3.7 | n.d. |

**Table S3 HPLC and headspace-GC analysis of volatile fatty acids and other metabolites in supernatant samples.**

*Limits of detection (LOD), µM: Acetaldehyde 1.1; ethanol 2.2; acetone 2.6; diacetyl 4.6; acetoin 62; α-ketoglutaric acid 68; glucose 111; pyruvic acid 114; lactic acid 333; formic acid 434; acetic acid 500.*


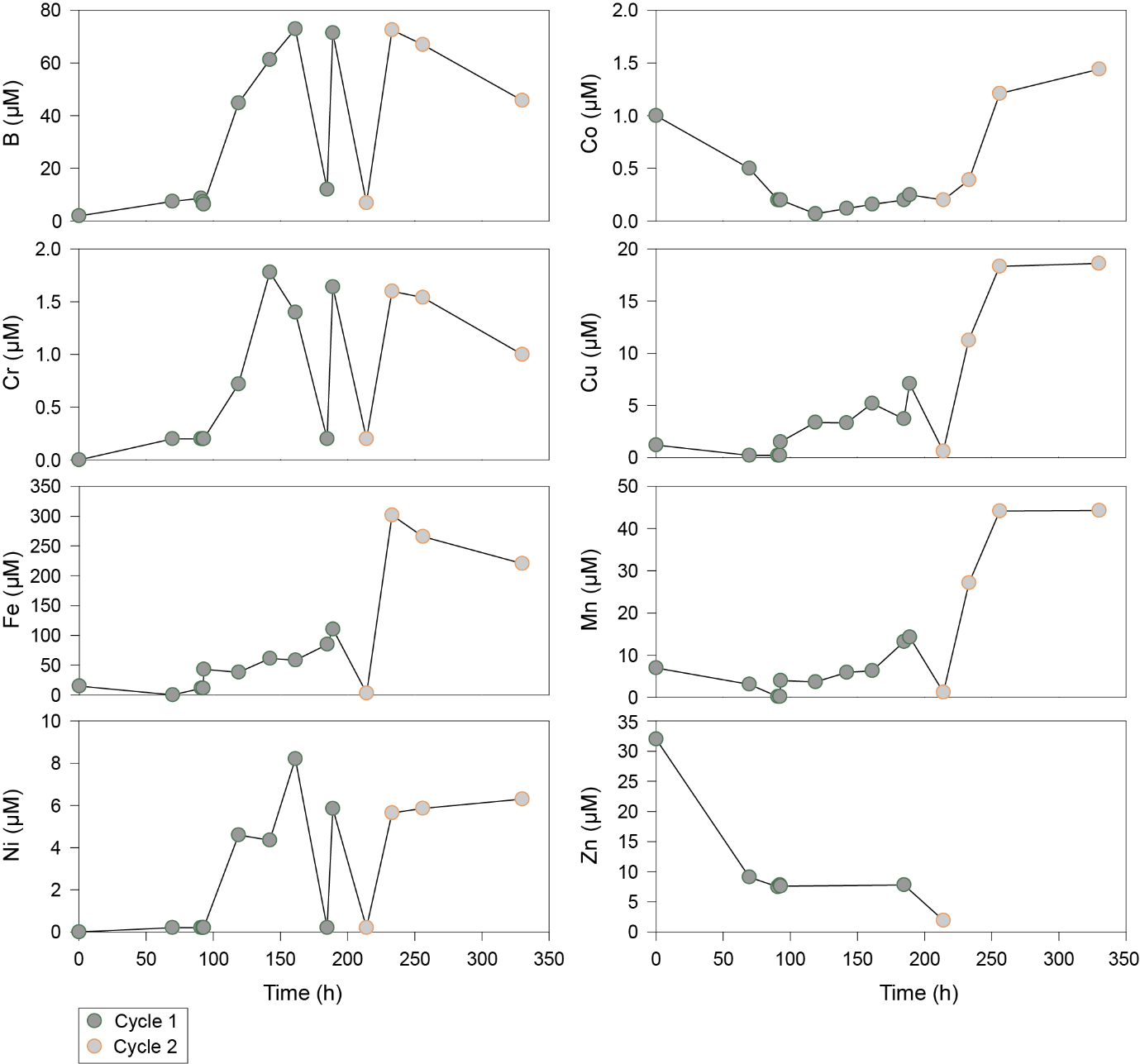


**Figure S5 ICP-MS of the reactor liquid throughout the run.** Liquid samples were taken during cycle 1 (dark green) and cycle 2 (orange) during the reactor run and analyzed by ICP-MS to determine the trace element concentration.
