# Supplementary material for: High cell density cultivation by anaerobic respiration
Source: Microb Cell Fact. 2024 Nov 25;23:320. doi: 10.1186/s12934-024-02595-8 (PMC11590539; doi:10.1186/s12934-024-02595-8)
Supplement: Supplementary file 5 — Additional file 5. E. Fed-batch 3. The file details the experimental setup of Fed-batch 3. Figure S6 shows the overview of the fed-batch including offline measurements [file 12934_2024_2595_MOESM5_ESM.docx]

Additional File E

High Cell Density Cultivation by Anaerobic Respiration

Marte Mølsæter Maråk^1^, Ricarda Kellermann, Linda Liberg Bergaust^1*^ and Lars Reier Bakken^1^.

*^1^Norwegian University for Life Sciences, Faculty of Biotechnology, Chemistry and Food Science*

*^*^Corresponding author:* [linda.bergaust@nmbu.no](mailto:linda.bergaust@nmbu.no)

Fed-batch 3

The reactor was set up equally to Fed-batch 2, except that the reactor was filled with 900 mL modified mineral base medium (M1 with TE-3). Anaerobically raised *P. denitrificans* was used as inoculum. Glucose was added to an initial concentration of 10 mM and a pulse of NO_3_^-^ was given by adjusting the pH setpoint by 0.2 pH units to 7.4. The experiment was run in four consecutive cycles, where each was initiated when OD_660_ was approximately 50 by replacing 90% of the reactor liquid with fresh sterile medium. TRES-3, KNO_3,_ and glucose were added several times during the run (Figure S6).


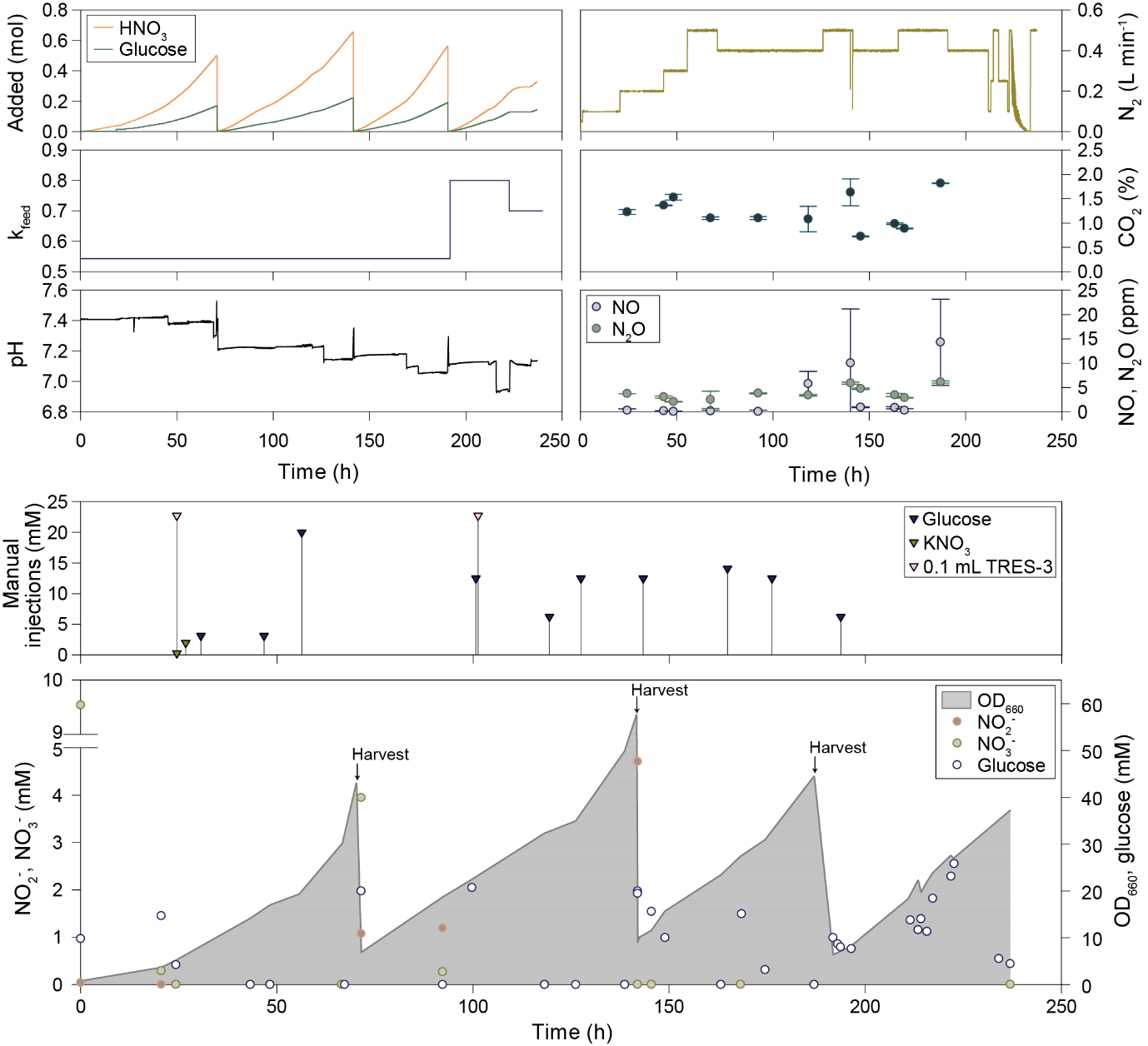


**Figure S6 Overview of Fed-batch 3.** The fed-batch spanned 240 hours divided into four cycles, each initiated by replacing most of the reactor liquid with fresh medium. The left panels depict the cumulative amounts of HNO_3_ and glucose added in each cycle, the k_feed_ value, and the measured pH. The right panels display the N_2_ sparging flow rate (L min^-1^) and the measured concentrations of CO_2_ (%), NO (ppm), and N_2_O (ppm) in the reactor headspace. The bottom panels illustrate the manual injections of glucose, KNO_3_, and TRES-3 and the measured concentration of NO_2_^-^, NO_3_^-^, and glucose in liquid samples, as well as OD_660_.
